# Directing Astroglia from the Cerebral Cortex into Subtype Specific Functional Neurons

**DOI:** 10.1371/journal.pbio.1000373

**Published:** 2010-05-18

**Authors:** Christophe Heinrich, Robert Blum, Sergio Gascón, Giacomo Masserdotti, Pratibha Tripathi, Rodrigo Sánchez, Steffen Tiedt, Timm Schroeder, Magdalena Götz, Benedikt Berninger

**Affiliations:** 1 Department of Physiological Genomics, Institute of Physiology, Ludwig-Maximilians University Munich, Munich, Germany; 2 Institute for Stem Cell Research, National Research Center for Environment and Health, Neuherberg, Germany; 3 Munich Center for Integrated Protein Science CiPSM, Munich, Germany; National Institutes of Health, United States of America

## Abstract

Astroglia from the postnatal cerebral cortex can be reprogrammed in vitro to generate neurons following forced expression of neurogenic transcription factors, thus opening new avenues towards a potential use of endogenous astroglia for brain repair. However, in previous attempts astroglia-derived neurons failed to establish functional synapses, a severe limitation towards functional neurogenesis. It remained therefore also unknown whether neurons derived from reprogrammed astroglia could be directed towards distinct neuronal subtype identities by selective expression of distinct neurogenic fate determinants. Here we show that strong and persistent expression of neurogenic fate determinants driven by silencing-resistant retroviral vectors instructs astroglia from the postnatal cortex in vitro to mature into fully functional, synapse-forming neurons. Importantly, the neurotransmitter fate choice of astroglia-derived neurons can be controlled by selective expression of distinct neurogenic transcription factors: forced expression of the dorsal telencephalic fate determinant neurogenin-2 (Neurog2) directs cortical astroglia to generate synapse-forming glutamatergic neurons; in contrast, the ventral telencephalic fate determinant Dlx2 induces a GABAergic identity, although the overall efficiency of Dlx2-mediated neuronal reprogramming is much lower compared to Neurog2, suggesting that cortical astroglia possess a higher competence to respond to the dorsal telencephalic fate determinant. Interestingly, however, reprogramming of astroglia towards the generation of GABAergic neurons was greatly facilitated when the astroglial cells were first expanded as neurosphere cells prior to transduction with Dlx2. Importantly, this approach of expansion under neurosphere conditions and subsequent reprogramming with distinct neurogenic transcription factors can also be extended to reactive astroglia isolated from the adult injured cerebral cortex, allowing for the selective generation of glutamatergic or GABAergic neurons. These data provide evidence that cortical astroglia can undergo a conversion across cell lineages by forced expression of a single neurogenic transcription factor, stably generating fully differentiated neurons. Moreover, neuronal reprogramming of astroglia is not restricted to postnatal stages but can also be achieved from terminally differentiated astroglia of the adult cerebral cortex following injury-induced reactivation.

## Abstract 2

Forced expression of single defined transcription factors can selectively and stably convert cultured astroglia into synapse-forming excitatory and inhibitory neurons.

## Author Summary

The brain consists of two major cell types: neurons, which transmit information, and glial cells, which support and protect neurons. Interestingly, evidence suggests that some glial cells, including astroglia, can be directly converted into neurons by specific proteins, a transformation that may aid in the functional repair of damaged brain tissue. However, in order for the repaired brain areas to function properly, it is important that astroglia be directed into appropriate neuronal subclasses. In this study, we show that non-neurogenic astroglia from the cerebral cortex can be reprogrammed in vitro using just a single transcription factor to yield fully functional excitatory or inhibitory neurons. We achieved this result through forced expression of the same transcription factors that instruct the genesis of these distinct neuronal subtypes during embryonic forebrain development. Moreover we demonstrate that reactive astroglia isolated from the adult cortex after local injury can be reprogrammed into synapse-forming excitatory or inhibitory neurons following a similar strategy. Our findings provide evidence that endogenous glial cells may prove a promising strategy for replacing neurons that have degenerated due to trauma or disease.

## Introduction

While exerting diverse functions within the brain parenchyma [1], astroglia are remarkable in that they also function as neural stem or progenitor cells in specific regions of the postnatal and adult brain [2], such as the ventricular subependymal zone [3] and the subgranular zone of the hippocampus [4],[5]. This raises the possibility that even astroglia from non-neurogenic regions such as the cerebral cortex may be reprogrammed towards neurogenesis when provided with the appropriate transcriptional cues. Indeed, we could previously show that astroglia from the early postnatal cerebral cortex can be reprogrammed in vitro towards the generation of neurons capable of action potential (AP) firing by a single transcription factor, such as Pax6 or its target, the pro-neural transcription factor neurogenin-2 (Neurog2) [6],[7]. These findings may open interesting avenues towards the potential activation of endogenous astroglia for neuronal repair of injured brain tissue.

However, several major obstacles remained to be overcome to fully exploit the potential of reprogrammed astroglia as an endogenous cellular source for neuronal repair. Firstly, reprogramming of astroglia towards neurons remained incomplete as the astroglia-derived neurons failed to establish a functional presynaptic output [7], an obvious hurdle towards functional repair that requires participation in a neural network. Secondly, given the lack of functional presynaptic output, we could not determine the neuronal subtype generated by the reprogrammed astroglial cells [7]. This raises the conceptual concern of whether neurons derived from astroglial cells in a given brain region may be restricted towards the generation of a respective neuronal subtype. During development of the forebrain in rodents, stem/progenitor cells in the dorsal telencephalon generate exclusively excitatory glutamatergic neurons, directed by Pax6 and Neurog1/2 [8]–[10], while stem/progenitor cells in the ventral telencephalon give rise primarily to inhibitory GABAergic neurons, governed by the fate determinants mammalian achaete-schute homolog 1 (Mash1) [11],[12] and Dlx1/2 [13]. Region-specific fate restriction also seems to apply for adult neural stem cells that are intrinsically specified towards the generation of distinct neuronal subtypes [14]. This implies that despite their multipotent nature in regard to generating different glial cell types and neurons, the subtype identity of the neurons generated from these stem cells is predetermined (see also [15]) and is not altered following transplantation [14]. This raises the important question of whether neuronal reprogramming of astroglia derived from the cerebral cortex, a region derived from the dorsal telencephalon, may be restricted towards the generation of glutamatergic neurons, or whether this region-specific bias could be overcome by forced expression of the appropriate neurogenic fate determinants. Such an ability to generate both glutamatergic and GABAergic neurons from astroglia may be a crucial step towards restoring a damaged or imbalanced neuronal network.

Towards this end, we first aimed at a more potent neuronal reprogramming by inducing higher and more persistent expression of neurogenic fate determinants in astroglial cells. This allowed us not only to obtain fully functional neurons that also establish synapses from astroglial cells in vitro but also to demonstrate that distinct neurogenic transcription factors, such as on the one hand Neurog2 and on the other Dlx2 alone or in combination with Mash1, can indeed instruct the selective generation of different neuronal subtypes, such as glutamatergic and GABAergic neurons, respectively. Moreover, we found that the reprogramming efficiency of postnatal cortical astroglia towards GABAergic neurons by Dlx2 could be enhanced by first expanding the astroglial cells under neurosphere conditions prior to forced expression of Dlx2. Given that following brain injury reactive astroglia from the adult cerebral cortex de-differentiate, resume proliferation, and can give rise to self-renewing neurospheres in vitro [16], we finally show that neuronal reprogramming and subtype specification are not restricted to postnatal stages but can also be achieved from adult cortical astroglia responding to injury.

## Results

### Postnatal Cortical Astroglia Reprogrammed by Forced Expression of Neurog2 Give Rise to Synapse-Forming Glutamatergic Neurons

Failure to establish a functional presynaptic compartment by astroglia-derived neurons may be due to an incomplete reprogramming [7]. Here, we hypothesized that stronger and more persistent expression of neurogenic fate determinants may be a prerequisite for a more complete reprogramming of astroglia towards synapse-forming neurons. We have previously shown that expression levels from LTR (long terminal repeat)-driven MMLV (Moloney Murine Leukemia Virus)-derived retroviral constructs, which we employed in previous studies, are only about 2–3-fold of the endogenous expression [6],[17]. Moreover, these viral vectors are prone to silencing [18] and we observed a severe decrease in Neurog2 or green fluorescent protein (GFP) reporter expression already 7–14 d after transduction [7],[19]. Thus, in order to overcome the limitations in synaptogenesis of neurons derived from reprogrammed astroglia, we examined the effect of stronger and more persistent expression of Neurog2 on neuronal reprogramming of astroglia from the cerebral cortex. We therefore subcloned Neurog2 into a self-inactivating retroviral vector driving gene expression under the control of a chicken beta-actin promoter (pCAG) optimized for long-term expression over months in the adult mouse brain [20]. Astroglia cultures were prepared from postnatal day 5–7 (P5–P7) cerebral cortex as described previously [7] and 1 wk later cells were passaged and subsequently transduced with a retroviral vector encoding Neurog2 and DsRed (pCAG-Neurog2-IRES-DsRed) or with a control virus encoding DsRed only (pCAG-IRES-DsRed). Consistent with a stronger and more persistent expression driven by the pCAG promoter, high levels of Neurog2 and DsRed protein were still detected at 5–6 wk following retroviral transduction of cortical astroglia (unpublished data).

**Figure 1:**
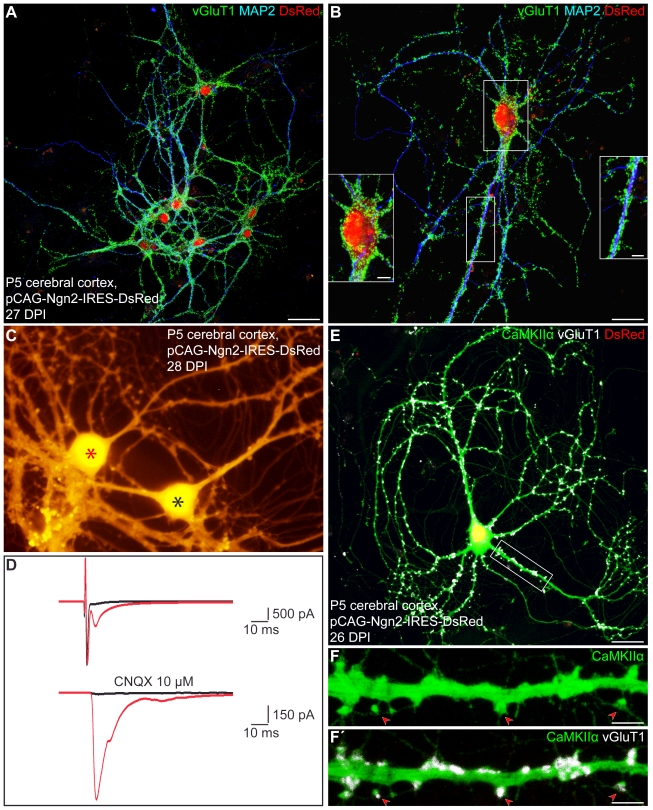
Postnatal cortical astroglia reprogrammed by forced expression of Neurog2 give rise to synapse-forming glutamatergic neurons. (A–B) Triple immunostaining for DsRed (red), MAP2 (blue), and vGluT1 (green) reveals that astroglia-derived neurons reprogrammed by Neurog2 exhibit a dense labelling of vGluT1-positive puncta outlining their soma and their MAP2-positive processes (27 DPI). (B) High magnification of a single astroglia-derived neuron illustrating the high density and the distribution of vGluT1-immunoreactive puncta. Insets show higher magnification views of the boxed areas. (C) The micrograph depicts two Neurog2-transduced cells exhibiting a neuronal morphology (28 DPI). The black and red asterisks mark the presynaptic and postsynaptic neurons recorded in panel (D), respectively. (D) Step-depolarisation of a presynaptic neuron evokes both an autaptic (upper red trace) and a monosynaptic response (delay 2 ms) in a nearby postsynaptic neuron (lower red trace). Both the synaptic and the autaptic responses were completely abolished in the presence of the AMPA/kainate receptor antagonist CNQX (10 µM; black traces), demonstrating the glutamatergic nature of the presynaptic neuron. (E) Triple immunostaining for DsRed (red), CaMKIIα (green), and vGluT1 (white). (F–F') High magnification views of the area boxed in (E) showing dendritic spines (red arrowheads) as revealed by CaMKIIα immunoreactivity (F), which are covered by vGluT1-positive puncta (F', red arrowheads), suggestive of sites of synaptic contact. Scale bars: A: 40 µm; B: 20 µm; insets in B: 5 µm; E: 26 µm; F–F': 5 µm.

In agreement with our previous observation on the high efficiency of neurogenesis from astroglia following forced Neurog2 expression, the vast majority of Neurog2-transduced astroglia had differentiated into βIII tubulin-positive, GFAP-negative neurons after 10 d in culture (Figure S1A and S1A'; 70.2%±6.3% at 9.8±3.1 days post-infection (DPI), 5 independent experiments, *n* = 1,022 DsRed-positive cells counted), in contrast to control retrovirus transduced cells (1.8%±1.8% of βIII tubulin-positive cells at 7.3±1.0 DPI, 3 independent experiments, *n* = 3,235 DsRed-positive cells counted). Time-lapse video microscopy revealed that the initial conversion of astroglia into neurons requires approximately 4 d, confirming previous results [7], and can occur at high efficiency (Video S1). By 2–3 wk post-transduction, neurons derived from Neurog2-transduced astroglia had acquired MAP2 immunoreactivity, indicative for dendritic maturation (Figure 1A and 1B). Most strikingly, immunostaining for the vesicular glutamate transporter 1 (vGluT1), present in synaptic vesicles within presynaptic terminals of glutamatergic neurons, revealed that the vast majority of astroglia-derived neurons exhibited a dense labelling with vGluT1-positive puncta outlining their soma and their MAP2-positive processes 4 wk post-infection with Neurog2 (Figure 1A and 1B, 85.4%±5.0% of DsRed-positive neurons at 26.3±2.2 DPI, *n* = 3 independent experiments, *n* = 170 DsRed-positive neurons counted). This was in pronounced contrast to the virtual absence of such staining upon transduction with the LTR-driven construct (pCLIG-Neurog2) as described previously [7] and also no vGluT1 immunoreactivity could be detected in astroglial cultures transduced with the control vector (unpublished data). Thus, these data suggest that astroglia reprogrammed with the pCAG-Neurog2-containing retroviral vector acquire a glutamatergic phenotype forming presynaptic specializations.

As vGluT1 immunoreactivity does however not allow to ascertain the neurotransmitter identity of an individual labelled neuron, as the vGluT1-positive puncta may arise from other neurons in the next set of experiments we assessed with single and pair electrophysiological recordings whether astroglial cells reprogrammed by Neurog2 indeed give rise to functional glutamatergic autapses or synapses after a period of 14–32 DPI. As shown in Figure 1D, suprathreshold step-depolarisation of a DsRed-positive neuron (i.e. presynaptic neuron, black asterisk, Figure 1C) resulted in an autaptic response in the stimulated neuron and an inward current in a nearby DsRed-positive neuron with a short delay typical of a monosynaptic connection (i.e. postsynaptic neuron, red asterisk, Figure 1C). In addition, the AMPA/kainate glutamate receptor antagonist CNQX completely abolished both the autaptic and the synaptic current, demonstrating the glutamatergic nature of the presynaptic neuron (Figure 1D). Among all the Neurog2-transduced astroglia-derived neurons recorded (*n* = 36, average age of cells: 24.6±0.9 DPI), 58.3% exhibited either glutamatergic autaptic connections onto themselves or glutamatergic synapses onto nearby neurons (Figure S2A). In none of the recordings from neurons derived from Neurog2-transduced astroglia was a GABAergic connection observed (Figure S2A). In accordance, cultures transduced with Neurog2 encoding retrovirus were devoid of any vesicular GABA transporter (vGaT) immunoreactivity (unpublished data). Thus, these data provide evidence that Neurog2 does not only induce a generic neuronal fate in postnatal astroglia but selectively elicits differentiation along the glutamatergic lineage, in exclusion of GABAergic neurogenesis. Consistent with the specification of postnatal astroglia towards a glutamatergic identity, forced expression of Neurog2 resulted in the induction of the T-box transcription factors Tbr2 (Figure S1B and S1B') in 20.7%±1.9% of the DsRed-positive cells at 4 DPI (*n* = 4 coverslips, *n* = 634 DsRed-positive cells counted) and Tbr1 (48.2% of DsRed-positive neurons at 7 DPI, *n* = 112 DsRed/βIII tubulin-double positive cells counted; Figure S1C and S1C') as shown previously [7], hence of two well characterised hallmarks of glutamatergic neurogenesis [21]. Moreover, by 4 wk of forced Neurog2 expression, astroglia-derived neurons expressed high levels of the forebrain glutamatergic neuron specific Ca^2+^/Calmodulin dependent kinase subunit IIα [22], accumulating at dendritic spine-like structures which were typically in opposition of vGluT1-positive presynaptic terminals (Figure 1E–1F').

**Figure 2:**
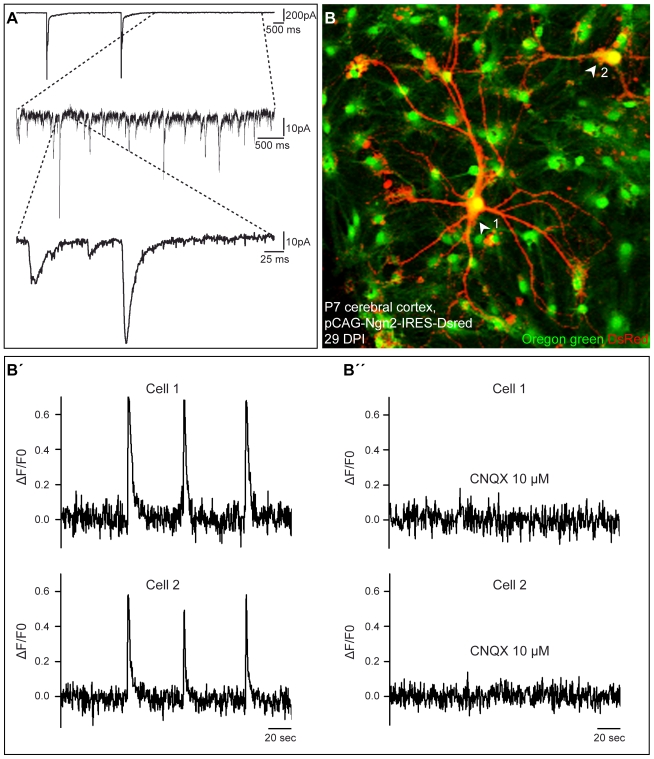
Neurons derived from Neurog2-reprogrammed astroglia generate spontaneous excitatory network activity. (A) Spontaneous synaptic activity recorded in cultures of Neurog2-reprogrammed astroglia at 28 DPI. The upper trace shows two large barrages of synaptic currents that are likely to underlie the spontaneous Ca^2+^ transients shown in (B–B”). The enlarged traces below show individual synaptic events during barrage-free periods of recording. (B–B”) Calcium imaging experiments performed at 29 DPI reveal self-driven network oscillations. (B) Micrograph depicting two DsRed-positive neurons (arrowheads) derived from Neurog2-reprogrammed astroglia recorded in (B'–B”). Cultures were incubated with the calcium-sensitive probe Oregon Green BAPTA1. (B') The graphs depict increases in Oregon Green fluorescence intensity over time (ΔF/F0) that reveal spontaneous and synchronous raises in free intracellular Ca^2+^ concentration in the two neurons shown in (B). (B”) These raises in Ca^2+^ concentration are completely abolished in the presence of CNQX (10 µM).

Consistent with the development of excitatory networks in Neurog2-reprogrammed astroglia cultures, we also observed the emergence of self-driven synaptic activity, resulting eventually in the occurrence of barrages of synaptic currents (Figure 2A). To monitor such self-driven activity, we performed calcium imaging experiments of neurons derived from Neurog2-reprogrammed astroglia. Figure 2B illustrates two neurons that exhibited spontaneous, recurrent, and synchronous Ca^2+^ transients (Figure 2B–2B”). These Ca^2+^ transients were completely abolished in the presence of CNQX (Figure 2B”). The majority of the DsRed-positive neurons that we analysed (63.8%, *n* = 47 imaged neurons, 3 independent experiments) exhibited Ca^2+^ transients at 14–43 d after transduction with Neurog2, thus indicating the high degree of incorporation of Neurog2-transduced astroglia into excitatory neuronal networks. These data clearly demonstrate that forced expression of Neurog2 driven by the pCAG retroviral vector is sufficient to instruct postnatal cortical astroglia to generate fully functional synapse-forming glutamatergic neurons.

### Genetic Fate Mapping Demonstrates Reprogramming of Postnatal Cortical Astroglia into Glutamatergic Neurons

**Figure 3:**
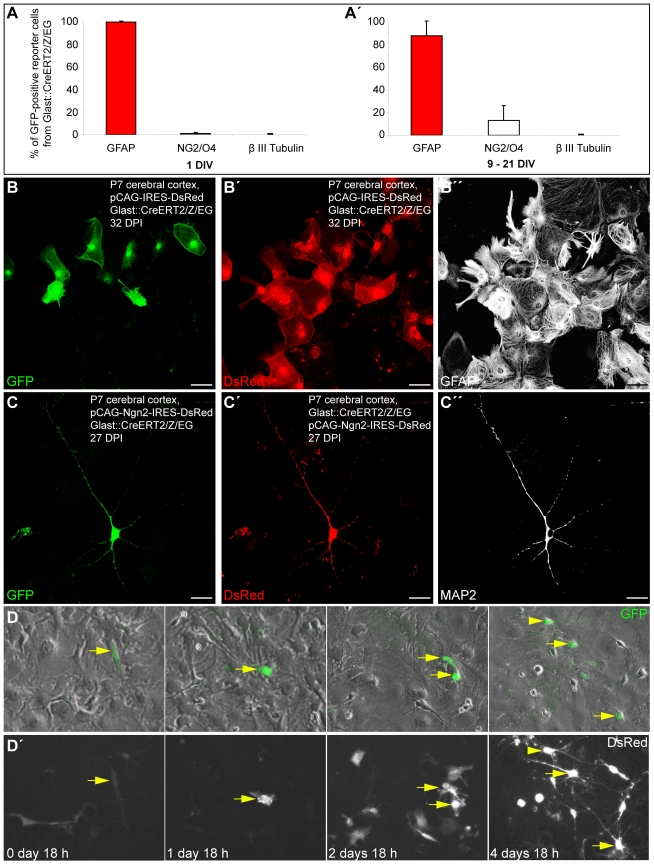
Fate-mapped astroglia from the postnatal cortex can be reprogrammed to generate neurons following forced expression of Neurog2. (A–A') The histograms show the percentage of GFP reporter-positive cells from GLAST::CreERT2/Z/EG mice immunoreactive for the astroglial marker GFAP, oligodendroglial markers NG2/O4, and the neuronal marker βIII tubulin 1 d after plating (A) and 9–21 d after plating (A'), respectively. (B–B”) Cortical astroglia transduced with control retrovirus remain in the glial lineage. (B) The micrograph depicts GFP-positive, fate-mapped astroglia derived from postnatally induced GLAST::CreERT2/Z/EG mice. (B'–B”) Micrographs of the same field of view as shown in (B) showing that fate-mapped astroglia transduced with a control retrovirus encoding DsRed only (pCAG-IRES-DsRed) exhibit a glial morphology and express GFAP (B”). (C) Representative example of a GFP-positive neuron, i.e. derived from a fate-mapped astroglia, prepared from the postnatal cortex of tamoxifen-induced GLAST::CreERT2/Z/EG mice. (C') DsRed expression in the same cell indicating forced expression of Neurog2. (C”) The same astroglia-derived neuron as shown in (C) and (C') is immunoreactive for the mature neuronal marker MAP2 (27 DPI). (D–D') Direct visualisation of Neurog2-induced reprogramming of fate-mapped astroglia by time-lapse video microscopy. (D) Sequence of bright field images, overlaid with GFP reporter fluorescence, depicting the same field of view over the time course of 5 d as indicated in the corresponding panels below in (D'). The arrow points to a reporter-positive cell that divided once giving rise to two daughter cells that subsequently underwent reprogramming into neurons. The arrowhead points to another fate-mapped reprogrammed cell that entered the field of view at a later time point. (D') Corresponding sequence of DsRed fluorescence images taken at the same time points as the bright field images shown in (D). Note the expression of DsRed (encoded by Neurog2-IRES-DsRed) in the fate-mapped cell (arrow). Note that besides fate-mapped cells, several other Neurog2-transduced cells also became reprogrammed into neurons. Scale bars: B–B”: 57 µm; C–C”: 26 µm.

In order to ascertain the astroglial nature of the cells that gave rise to functional glutamatergic synapses following reprogramming by Neurog2, we took advantage of a transgenic mouse line in which GFP expression can be induced in astroglia and is maintained in their progeny. Heterozygous mice in which the expression of a tamoxifen-inducible Cre recombinase is driven by the astroglia specific L-glutamate/L-aspartate transporter promoter (GLAST::CreERT2) [23] were crossed to a reporter mouse line (Z/EG) [24] to generate double heterozygous mutants (GLAST::CreERT2/Z/EG) that were used in the present study. Cre-mediated recombination of the reporter locus was induced via tamoxifen administration from postnatal day 2 (P2) until sacrifice (P5–P7). Astroglia cultures were prepared as described above and, 1 wk later, cells were passaged onto glass coverslips. The vast majority of GFP reporter-positive cells were immunoreactive for GFAP (98.7%±0.7%) at 1 d after plating, with few cells being positive for the oligodendroglial markers NG2/O4 (1.2%±0.7%) and none (0.1%±0.1%) for the neuronal marker βIII tubulin (Figure 3A; *n* = 3 independent experiments, *n* = 1,560 GFP-positive cells counted). These data indicate that, under our culture conditions, most reporter-positive cells at the time of transduction possess an astroglial identity. These cells largely remain within their astroglial lineage (86.9%±12.7% of GFP-positive cells expressing GFAP) when analysed at later stages (Figure 3A'; *n* = 4 independent experiments, *n* = 1,363 GFP-positive cells counted; 9–21 d following plating). We noted, however, a slight increase in the number of NG2/O4-positive cells (13.0%±12.8%), likely due to the expansion of few reporter-positive clones of oligodendrocyte precursors. Also at later stages reporter-positive cells did not give rise to βIII tubulin-positive neurons (0.1%±0.1%; Figure 3A').

To determine the identity of fate-mapped astroglial cells following retroviral transduction, we performed immunostaining for GFP (identifying cells of astroglial origin), DsRed (identifying transduced cells), and either βIII tubulin, MAP2, or GFAP (identifying neuronal and astroglial cells, respectively). Notably, the stochastic infection of the subset of genetically recombined cells results in a limited number of double-targeted cells. When cultures of adherent astroglia were transduced with the control retrovirus encoding DsRed only, fate-mapped astroglial cells co-expressing GFP and DsRed remained in the glial lineage, as revealed by their astroglial morphology and GFAP expression 1 mo after transduction (Figure 3B–3B”). In sharp contrast, when cultures of tamoxifen-induced astroglia were transduced with the new retrovirus encoding Neurog2 and DsRed, most GFP/DsRed-double-positive fate-mapped astroglia were reprogrammed into neurons expressing the neuronal markers βIII tubulin and MAP2 (67.3%±12.7% among GFP/DsRed-double positive cells at 8.0±1.0 DPI, *n* = 3 independent experiments, *n* = 217 double-positive cells counted; Figure 3C–3C”). Single cell tracking of GFP-reporter positive cells following Neurog2-transduction allowed the direct visualisation of the glia-to-neuron conversion of fate-mapped cells over the time course of 5 d (Figure 3D and 3D'; Video S1 and Video S2).

**Figure 4:**
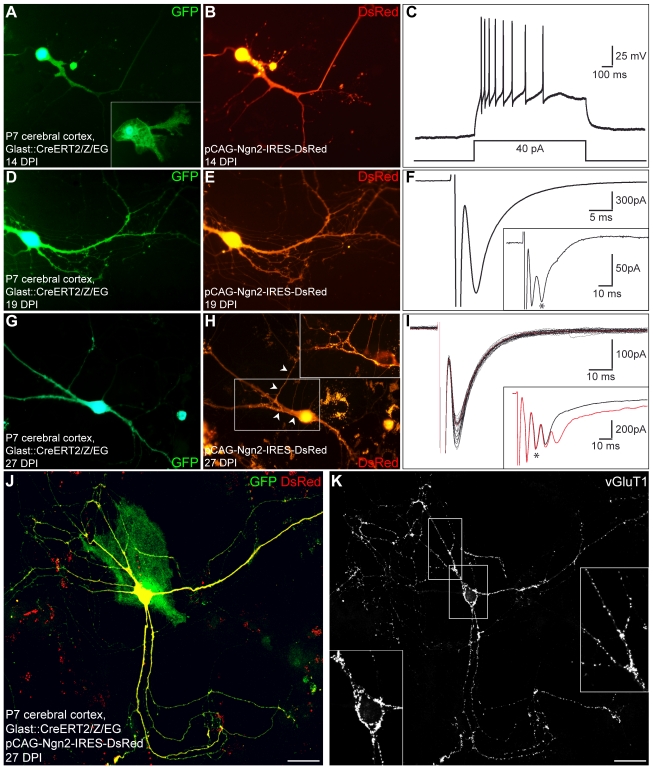
Fate-mapped cortical astroglia reprogrammed by forced expression of Neurog2 establish functional synaptic connections. (A) Fluorescence micrograph depicting a GFP-positive neuron derived from a fate-mapped astroglia, prepared from postnatal cortex of tamoxifen-induced GLAST::CreERT2/Z/EG mice, 14 d after transduction with Neurog2-DsRed. The inset shows control untransduced GFP-positive astroglia. (B) DsRed expression in the same cell indicating forced expression of Neurog2. (C) Step-current injection into the cell shown in (A) and (B) results in repetitive firing of action potentials. (D–E) Fluorescence micrographs depicting another GFP-positive neuron, derived from a fate-mapped astroglia, following Neurog2-induced reprogramming 19 d after transduction. (F) Step-depolarisation at 1 Hz of the neuron shown in (D) and (E) evokes an autaptic response exhibiting a short decay time (90%–10%) typical of glutamatergic synaptic transmission (5.2 ms). The inset shows a single response evoked at 0.05 Hz revealing both an autaptic and a polysynaptic response (asterisk) due to recruitment of other neurons in the cultured network, indicating the excitatory nature of the fate-mapped reprogrammed neuron. (G–H) Another example of a GFP-positive neuron derived from a fate-mapped astroglia following Neurog2-induced reprogramming 27 DPI. The inset in (H) shows an enlargement of the boxed area revealing a DsRed-positive axon (arrowheads) originating from another DsRed-positive neuron and meandering along the dendrites and the soma of the recorded cell, after the patch pipette had been withdrawn and the cell died. (I) Step-depolarisation of the neuron shown in (G) and (H) evokes a sequence of both autaptic and polysynaptic responses. The black traces show individual autaptic responses observed in isolation when evoked at 1 Hz to eliminate polysynaptic components (the average trace is shown in red). The autapse exhibited a short decay time (90%–10%) typical of glutamatergic synaptic transmission (7.7 ms). The inset shows two individual responses evoked at 0.05 Hz (black and red traces) revealing both autaptic and polysynaptic responses (asterisk) due to recruitment of other neurons in the cultured network, indicating the excitatory nature of the fate-mapped reprogrammed neuron. (J–K) Expression of the vesicular glutamate transporter 1 (vGluT1) by fate-mapped postnatal astroglia reprogrammed by Neurog2. (J) Micrograph depicting a GFP and DsRed double-positive neuron derived from a fate-mapped astroglia, 27 d after transduction with a retrovirus encoding Neurog2 and DsRed. Note the DsRed-negative fate-mapped astrocyte. (K) Immunocytochemistry for vGluT1 reveals that the fate-mapped astroglia-derived neuron reprogrammed by Neurog2 exhibits a dense labelling of vGluT1-positive puncta outlining its cell body and dendrites. The insets show higher magnification views of the soma and dendrites illustrating the punctuate staining for vGluT1. Scale bars: J-K: 30 µm.

Perforated patch clamp recordings of these fate-mapped astroglia-derived cells reprogrammed by Neurog2 revealed their functional neuronal identity as these cells fired APs following step-current injection in current clamp (*n* = 8; Figure 4A–4C). In the next set of experiments, we assessed whether neurons derived from fate-mapped astroglia could give rise to functional glutamatergic autapses (Figure 4D–4I). Step-depolarisation of GFP/DsRed-double-positive neurons at 0.05 Hz evoked a sequence of both autaptic and polysynaptic components (2 out of 8 cells recorded) consistent with the excitatory nature of the recorded neurons (average age of the cells: 18.1±2.2 DPI; Figure 4D–4I, insets), while at higher stimulation frequency (1 Hz) the autaptic component with a short decay time typical of glutamatergic synaptic transmission [25] could be observed in isolation (Figure 4F and 4I). Consistent with their glutamatergic nature, fate-mapped astroglia reprogrammed by forced expression of Neurog2 also exhibited a dense labelling of vGluT1-positive puncta (Figure 4J and 4K). These data clearly demonstrate that Neurog2 instructs fate-mapped astroglia from the postnatal cerebral cortex to acquire a glutamatergic identity.

### Cell Division Is Not a Sine Qua Non Condition for Reprogramming of Astroglia by Neurog2

**Figure 5:**
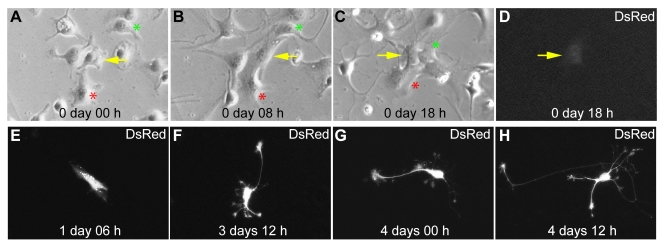
Cell division is not a requirement for neuronal reprogramming of postnatal astroglia by forced expression of Neurog2. Time-lapse sequence of an astroglia culture transfected with a retroviral plasmid encoding Neurog2 and DsRed. The respective time points are shown in the individual panels (from 0 d 00 h to 4 d 12 h). (A–C) Bright field micrographs show high magnification views of an astroglial culture during the first 18 h of continuous time-lapse imaging. The arrow marks a cell that was subsequently found to be transfected (D–H). The asterisks mark other astroglial cells in the field of view for orientation. (D–H) DsRed-fluorescence micrographs depicting the same cell marked by the arrow in (A–C). Note the onset of weak DsRed expression at 18 h (D). The fluorescence micrographs at subsequent time points show the progressive reprogramming of the transfected astrocyte into a neuron which is completed by 4 d 12 h. Note that during the entire imaging period the cell giving rise to a neuron did not undergo cell cycle division.

Given that our reprogramming strategy is based on retrovirally mediated expression of neurogenic fate determinants, only cells undergoing cell division will be targeted. In order to examine whether cell division is required for fate conversion to occur, we assessed whether neuronal reprogramming can be also achieved when the Neurog2 and DsRed encoding plasmid is delivered to the postnatal astroglia by transfection, i.e. a gene transfer strategy which does not select for dividing cells, and tracked single transfected cells by time-lapse video microscopy. Transfection with the Neurog2 encoding plasmid resulted in a similar degree of reprogramming after 4 d (14 cells out of 17, Figure 5) as obtained after retroviral transduction. Of note, in four cases neurons were generated directly from single astrocytes without a prior cell division (Figure 5 and Video S3). Thus direct lineage conversion can occur in the absence of cell division.

### Dlx2 Directs Postnatal Cortical Astroglia towards Acquiring a GABAergic Identity

Based on our finding that forced expression of Neurog2 can selectively drive cortical astroglia towards the generation of functional and synaptically integrated glutamatergic neurons, we next asked whether cortical astroglia may also be directed towards distinct neuronal subtypes. In particular, we asked whether neuronal fate determinants known to instruct the genesis of GABAergic neurons during embryonic development may be sufficient to exert a similar effect on postnatal astroglia. As the homeobox transcription factor Dlx2 is one of the key factors involved in GABAergic neuron specification in the developing ventral telencephalon [13] and in adult neurogenesis [26], we examined whether forced expression of Dlx2 is also sufficient to induce a neuronal and possibly a GABAergic fate in cortical astroglia. To test this, astroglia cultures from P5–P7 cortex of C57BL/6J or GLAST::CreERT2/Z/EG mice were transduced with the same high-expressing retrovirus encoding in this case Dlx2 and DsRed (pCAG-Dlx2-IRES-DsRed), and cells were immunostained for GFP (to identify cells of astroglial origin), DsRed (to identify Dlx2-transduced cells), and the neuronal markers βIII tubulin or MAP2 after various differentiation time periods in culture.

**Figure 6:**
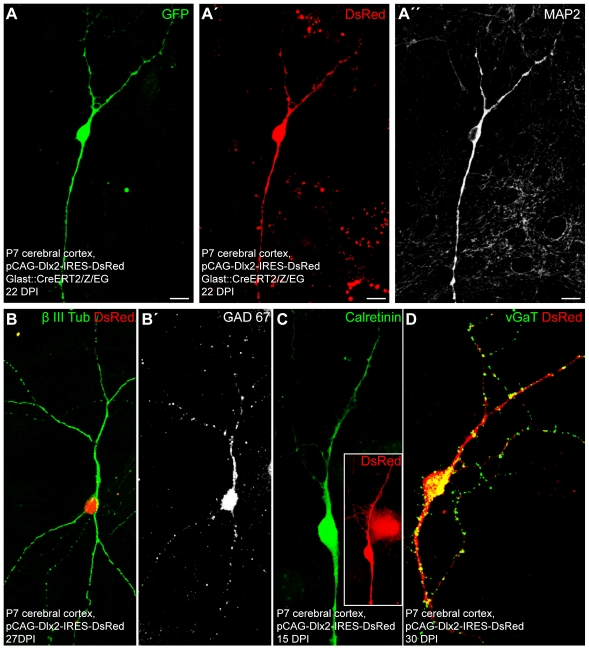
Fate-mapped astroglia from the postnatal cortex can be reprogrammed to generate neurons by forced expression of Dlx2. (A) Representative example of a GFP-positive neuron derived from a fate-mapped cortical astroglia, originating from postnatally induced GLAST::CreERT2/Z/EG mice, reprogrammed by forced expression of Dlx2 and shown at 22 DPI. (A') DsRed expression in the same cell indicating forced expression of Dlx2. (A”) The same astroglia-derived neuron as shown in (A) and (A′) is immunoreactive for the mature neuronal marker MAP2. (B–B') Triple immunostaining of a Dlx2-transduced cell positive for the neuronal marker βIII tubulin (B, green), DsRed (B, red), and GAD67 (B', white). (C) Double immunostaining of a Dlx2-transduced cell positive for DsRed (inset, red) and calretinin (green). (D) Double immunostaining for DsRed (red) and vGaT (green) showing an astroglia-derived neuron reprogrammed by forced expression of Dlx2 at 30 DPI, that exhibits a dense labelling of vGaT-positive puncta outlining its soma and processes. Scale bars: A–A″: 10 µm.

**Figure 7:**
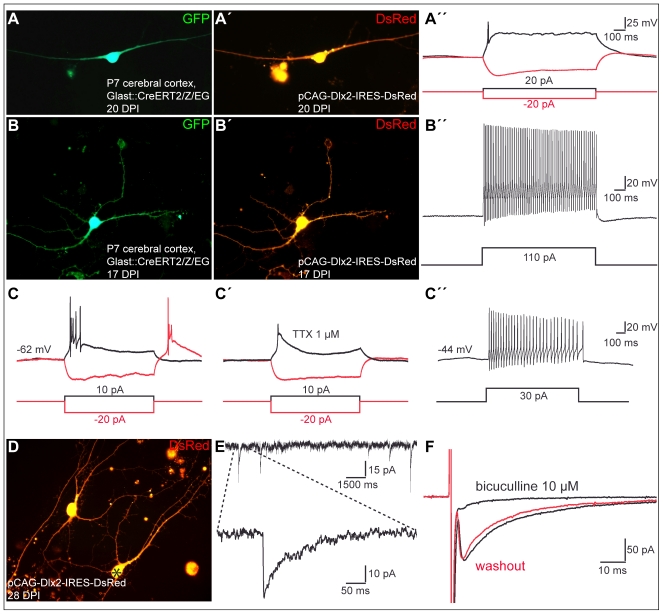
Postnatal cortical astroglia reprogrammed by forced expression of Dlx2 give rise to synapse-forming GABAergic neurons. (A) Fluorescence micrograph depicts a GFP-positive neuron derived from a fate-mapped astroglia, prepared from postnatal cortex of tamoxifen induced GLAST::CreERT2/Z/EG mice that has been reprogrammed by forced expression of Dlx2. (A') DsRed expression in the same cell at 20 DPI indicates prior reprogramming by Dlx2. (A”) Step-current injection in current clamp in the cell shown in (A) and (A') results in a single action potential. (B) Fluorescence micrograph depicts a GFP-positive neuron derived from a fate-mapped astroglia, prepared from postnatal cortex of tamoxifen induced GLAST::CreERT2/Z/EG mice that has been reprogrammed by forced expression of Dlx2. (B') DsRed expression in the same cell at 17 DPI indicates prior reprogramming by Dlx2. (B”) Step-current injection in current clamp in the cell shown in (B) and (B') results in repetitive firing of action potentials. Note the high action potential firing rate undergoing little adaptation (approximately 80 Hz). (C–C”) Traces show recordings from a Dlx2-reprogrammed cell classified as a low-threshold burst-spiking neuron. (C) Depolarizing current injection at a holding potential of −62 mV resulted in a burst discharge, while a hyperpolarizing current injection (red trace) induced a rebound burst following the relief from hyperpolarisation. (C') In the presence of TTX, burst-spiking was suppressed revealing a long-lasting spike, presumably mediated by low-threshold calcium channels. (C”) Depolarizing current injection at a more positive holding potential (−44 mV) resulted in repetitive firing instead of a burst discharge typical of low-threshold burst spiking interneurons. (D) Fluorescence micrograph depicting neurons derived from Dlx2-transduced astroglia at 28 DPI. (E) The graph depicts spontaneous synaptic events recorded from the astroglia-derived neuron marked by the asterisk in (D). The enlarged trace shows a single event displaying a slow decay time typical of GABAergic synaptic events. (F) Step-depolarisation in voltage clamp of a Dlx2-reprogrammed astroglia evokes a GABAergic autaptic response at 30 DPI (lower black trace), which is blocked by the GABA_A_ receptor antagonist bicuculline (10 µM, upper black trace). The red trace shows the recovery of the evoked autaptic response after washout of bicuculline.

**Figure 8:**
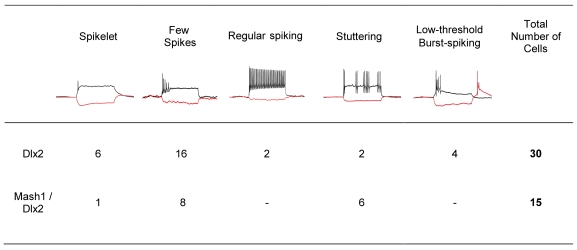
Classification of action potential firing patterns of astroglia reprogrammed by forced expression of Dlx2 alone or Mash1 and Dlx2 in combination. Note that combined expression of Mash1 and Dlx2 results in a higher fraction of neurons exhibiting more mature interneuron firing patterns compared to Dlx2 alone.

Upon forced expression of Dlx2, a substantial number of postnatal cortical astroglia were redirected towards a neuronal identity as revealed by βIII tubulin or MAP2 expression (35.9%±13.0% at 10.7±2.0 DPI, *n* = 3 independent experiments, *n* = 392 DsRed-positive cells counted). Notably, however, the efficacy of neurogenesis elicited by Dlx2 was significantly lower than the one elicited by Neurog2 (see above). Fate-mapping analysis confirmed the astroglial nature of the cells reprogrammed by forced expression of Dlx2 as observed 22 DPI (Figure 6A–6A”). Next, to confirm the neuronal identity of the astroglia-derived cells, we performed patch clamp recordings. All the cells expressing Dlx2 and exhibiting a neuronal morphology, that we recorded, were capable of AP firing in response to step current injection (*n* = 33). In particular, this also held true for GFP-positive neurons originating from fate-mapped astroglia that had been reprogrammed by Dlx2 (*n* = 9; Figure 7A–7C”). Notably, neurons derived from Dlx2-transduced astroglia exhibited distinct firing patterns, with most of them revealing immature characteristics (single to few spikes, 22 out of 30 cells recorded) (Figure 7A” and Figure 8). The eight remainder cells exhibited firing patterns which could be classified into three categories [27],[28], namely regular, stuttering, and low-threshold burst spiking (Figure 7B”–7C” and Figure 8), suggestive of the maturation into distinct types of non-fast spiking interneurons [29]. Similarly, the majority of fate-mapped astroglia reprogrammed by Dlx2 (7 out of 9 cells recorded) exhibited immature firing patterns (Figure 7A–7A”), while 2 out of 9 fate-mapped cells developed more mature interneuron-like firing (Figure 7B–7C”). Consistent with the generation of regular- and burst-spiking interneurons [29], we observed calretinin immunoreactivity in a small subset of the Dlx2-expressing cells (Figure 6C), while no parvalbumin immunoreactivity could be detected.

The predominant appearance of immature firing patterns, however, suggests an overall hampered maturation of Dlx2-reprogrammed astroglia. Accordingly, astroglia-derived neurons reprogrammed by Dlx2 exhibited much higher input resistance values than Neurog2-derived neurons after the same time in culture (Figure S2B and S2C; 2,319.2±187.9 MΩ at 26.0±1.4 DPI (*n* = 26) versus 1,111.8±211.1 MΩ at 23.7±1.5 DPI (*n* = 20), Dlx2 versus Neurog2, respectively). Surprisingly, the high input resistance of Dlx2-expressing neurons did not decrease but even slightly increased with time in culture (Figure S2B; 2,786.4±440.3 MΩ at 35.9±1.2 DPI (*n* = 7)), while in the case of Neurog2-reprogrammed astroglia input resistance decreased over time (608.6±125.0 MΩ at 26.8±1.2 DPI; *n* = 14; Figure S2C). Taken together, these data show that some postnatal cortical astroglia can be redirected by forced expression of Dlx2 towards a neuronal identity; however, in sharp contrast to the progressive maturation of Neurog2-transduced cells, most of the astroglia-derived neurons reprogrammed by Dlx2 remain in a rather immature state, suggesting a comparatively less efficient reprogramming by Dlx2.

Next we assessed whether some of these relatively immature neurons derived from Dlx2-reprogrammed astroglia may nevertheless establish functional autaptic or synaptic connections. We first performed immunocytochemistry for vGluT1 and for vGaT, the latter known to be expressed in synaptic vesicles located in presynaptic terminals of GABAergic neurons. In sharp contrast to reprogramming by Neurog2, astroglia-derived neurons reprogrammed by Dlx2 were devoid of vGluT1 immunoreactivity (unpublished data), but some of them (33.7%±3.6% at 22.0±0.6 DPI, *n* = 339 DsRed-positive neurons counted; *n* = 3 independent experiments) were found to exhibit labelling of vGaT-positive puncta outlining both their soma and their processes (Figure 6D). In addition, a small subset of DsRed-positive neurons exhibited GAD67 immunoreactivity (Figure 6B and 6B'). These findings therefore suggest that Dlx2 induces a GABAergic identity in the reprogrammed astroglia. Consistent with an interneuron phenotype, we could also record in 9 out of 33 neurons spontaneous synaptic currents exhibiting a slow decay time, characteristic of GABAergic synaptic events (Figure 7D and 7E). Finally, in few cases, step-depolarisation in voltage clamp evoked an autaptic response of the stimulated neuron (6.1% of the DsRed-positive neurons recorded, *n* = 33, age of the cells: 26.9±1.4 DPI; Figures 7F and S2A). In accordance with the above data, these autaptic responses exhibited slow decay time kinetics and were abolished by the GABA_A_ receptor antagonist bicuculline (Figure 7F), thus demonstrating the GABAergic nature of these autapses.

Taken together, these data strongly indicate that forced expression of Dlx2, in sharp contrast to Neurog2, can induce the reprogramming of astroglia from the postnatal cortex towards a GABAergic phenotype. However, whereas Neurog2 redirected the majority of astroglia towards functional glutamatergic neurons, only few astroglial cells reprogrammed by Dlx2 differentiated into fully functional, GABAergic neurons (58% versus 6%, respectively), thus indicating that Dlx2-induced reprogramming remains partial in most of the cells.

**Figure 9:**
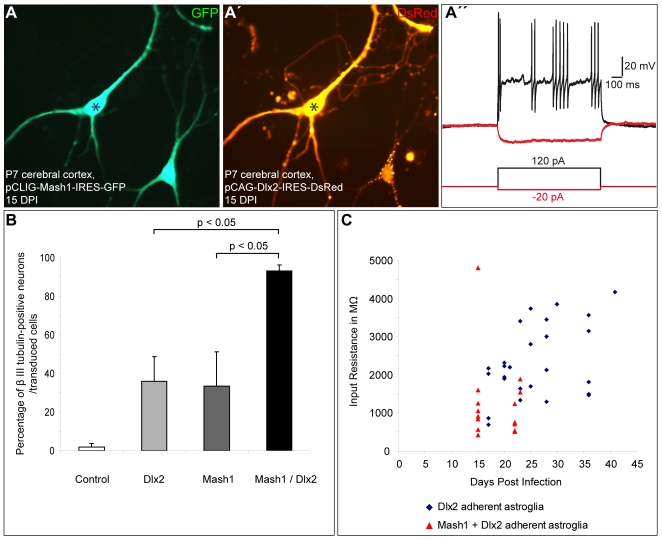
Co-expression of Mash1 and Dlx2 promotes neurogenesis and maturation following reprogramming. (A–A') Fluorescence micrographs depict two cells double-positive for GFP (A) and DsRed (A') indicating co-expression of Mash1 (Mash1-IRES-GFP) and Dlx2 (Dlx2-IRES-DsRed), respectively. (A”) Depolarizing current injection into the neuron marked by the asterisk in (A) and (A') revealed a firing pattern classified as stuttering. (B) The histogram shows the percentage of βIII tubulin-positive neurons amongst all single or co-transduced astroglial cells. Note the significant enhancement of neurogenesis following co-expression of Mash1 and Dlx2. (C) The graph plots the input resistance values over time (MΩ) of astroglia-derived neurons reprogrammed by Dlx2 alone or Mash1 and Dlx2 in combination. Note that the co-expressing cells exhibit lower input resistances suggestive of a faster pace of maturation.

Since we have previously shown that Mash1, a transcription factor located up-stream of Dlx2 in the interneuron fate specification [30], the direct targets of which overlap only partially with that of Mash1 [31], can also reprogram postnatal astroglia towards neurogenesis [7], we tested whether co-expression of these two transcription factors further promote neurogenesis and subsequent interneuron differentiation of reprogrammed astroglia [32]. Consistent with previous data [7], 33.5%±17.8% of astroglia expressing Mash1 alone developed into βIII tubulin-positive neurons (Figure 9B, *n* = 3 independent experiments, *n* = 226 DsRed-positive cells counted at 10.7±2.0 DPI). In contrast, co-expression of Mash1 and Dlx2 significantly augmented neurogenesis from postnatal astroglia (93.0%±3.1% of βIII tubulin-positive neurons amongst DsRed-positive cells, *n* = 3 independent experiments, *n* = 548 DsRed-positive cells counted at 10.7±2.0 DPI; Figure 9B), indicating that these two factors indeed act synergistically. Moreover, compared to cells expressing Dlx2 alone, Mash1/Dlx2 co-expressing neurons exhibited lower input resistance values (1,237.5±278.8 MΩ at 18.4±1.0 DPI; *n* = 15; Figure 9C). Consistent with a more mature status, a higher proportion of Mash1/Dlx2 co-expressing neurons exhibited specific interneuronal firing patterns (6 out of 15 cells recorded, Figure 8 and Figure 9A–9A”) compared to Dlx2 (8 out of 30 cells recorded, Figure 8). Despite this enhanced degree of differentiation, none of the recorded cells co-expressing Mash1 and Dlx2 showed an autaptic response (Figure S2A, *n* = 15).

Taken together, our data provide evidence that postnatal astroglia from the cerebral cortex can be driven towards the generation of interneurons with distinct functional properties by forced expression of Dlx2 or Dlx2 in combination with Mash1.

### Expansion of Postnatal Cortical Astroglia under Neurosphere Conditions Increases the Efficiency of Reprogramming towards a GABAergic Neuronal Subtype

**Figure 10:**
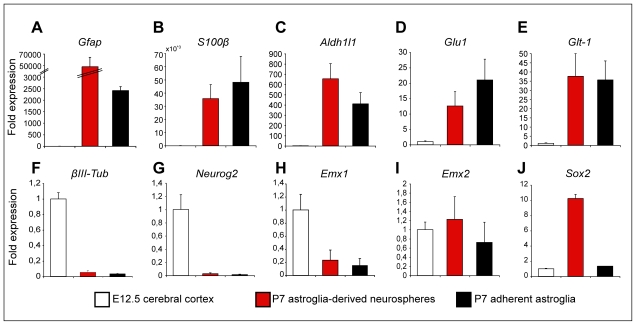
Expression analysis of molecular markers shows that astroglia cultured under neurosphere conditions keep an astroglial signature, while up-regulating Sox2. Cells from the E12.5 cerebral cortex (white bars), astroglia cultured under adherent conditions (7 d, black bars), and astroglia cultured under neurosphere conditions (7 d, red bars) were compared for the expression of several genes by quantitative RT-PCR. The expression of the mRNAs encoding GFAP (A), S100β (B), Aldh1L1 [37] (C), Glu1 (D), Glt-1 (E), βIII tubulin (F), Neurog2 (G), Emx1 (H), Emx2 (I), and Sox2 (J) was normalized to the respective mRNA amount estimated for E12.5 cerebral cortex tissue and plotted as fold expression.

Next, we examined whether a complete and more efficient reprogramming of astroglia towards synapse-forming functional GABAergic neurons may be achieved by first expanding astroglial cells as neurospheres in presence of mitogens that on the one hand promote de-differentiation of astroglia and on the other hand up-regulate fate determinants normally involved in the generation of GABAergic neurons in the telencephalon, such as Mash1 [33]–[35]. We therefore cultured postnatal astroglial cells as neurospheres before transducing the astroglia-derived neurosphere cells with Dlx2. It has been previously shown that early postnatal cortical astroglial cells can give rise to neurospheres until P11 [36]. Cortical tissue from P5–P7 C57BL/6J, GLAST::CreERT2/Z/EG, or hGFAP-GFP mice was cultured as neurospheres under non-adherent conditions in serum-free medium and in the presence of EGF/FGF2, and 1 wk later, astroglia-derived neurosphere cells were passaged and subsequently transduced with retrovirus encoding either Dlx2-DsRed or Neurog2-DsRed, and were allowed for differentiation. The astroglial origin of the neurosphere founder cells was confirmed by culturing single GFP-positive cells derived from the postnatal cortex of hGFAP-GFP mice which gave rise to neurospheres (Figure S3A–S3D). Moreover, quantitative RT-PCR demonstrated that during expansion in EGF/FGF2 neurosphere cells expressed mRNAs for different astroglial markers, such as the specific pan-astrocyte marker Aldh1L1 [37], at similarly high levels as adherent astroglia after 1 wk in culture, and the mRNA encoding GFAP at even higher levels (Figure 10A–10E). In contrast, no βIII tubulin mRNA could be detected (Figure 10F). Likewise, similar to adherent astroglia neurosphere cells did not express detectable levels of endogenous Neurog2 mRNA (Figure 10G). These data support the notion that during the expansion in EGF/FGF2, neurosphere cells have an astroglial character.

**Figure 11:**
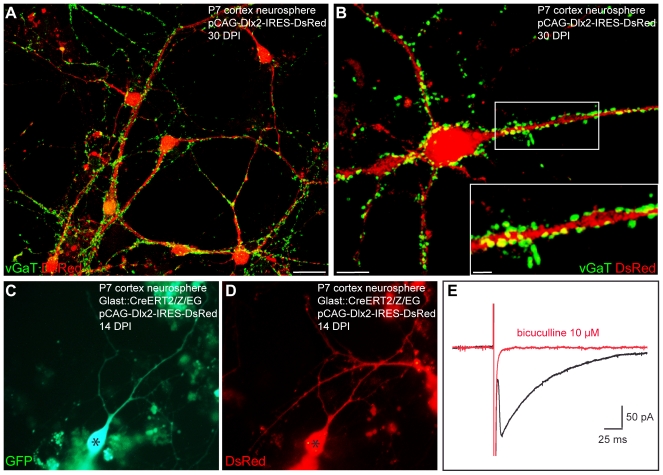
Reprogramming of postnatal astroglia expanded as neurosphere cells into functional GABAergic neurons following forced expression of Dlx2. (A) Postnatal cortical astroglia were first expanded as neurospheres and subsequently transduced with a retrovirus encoding Dlx2 and DsRed. Double immunocytochemistry for DsRed (red) and vGaT (green) reveals that astroglia-derived neurosphere cells reprogrammed by Dlx2 exhibit a dense labelling of vGaT-positive puncta outlining their soma and their dendrites, indicating that Dlx2-reprogrammed neurons acquire a GABAergic identity (30 DPI). (B) High magnification view of a single astroglia-derived neurosphere cell transduced with Dlx2 illustrating the massive expression and the distribution of vGaT-positive puncta. The inset shows a high magnification view of the boxed area. (C–D) The fluorescence micrographs depict a fate-mapped, GFP-positive neuron (C, asterisk) co-expressing DsRed at 14 DPI (D, asterisk), indicating forced expression of Dlx2. (E) Step-depolarisation of the neuron shown in (C) and (D) evokes a GABAergic autaptic response (black trace) that is blocked by bicuculline (10 µM, red trace). Scale bars: A: 20 µm; B: 7 µm; Inset in B: 2 µm.

In contrast to adherent astroglia cultures, 94.7%±0.3% (*n* = 3 independent experiments, *n* = 644 DsRed-positive cells counted) of the astroglia-derived neurosphere cells transduced with Dlx2 differentiated into MAP2-positive neurons (Figure S4A and S4C). Strikingly, even at younger stages in culture (20.1±1.9 DPI), Dlx2-expressing neurons derived from neurosphere cells exhibited substantially lower input resistances (1,266.4±294.3 MΩ, *n* = 7) compared to Dlx2-reprogrammed adherent astroglia recorded at 4 wk in culture (2,319.2±187.9 MΩ at 26.0±1.4 DPI, *n* = 26; Figure S2B), indicative of a more advanced neuronal maturation. Immunostaining for vGaT revealed a dense labelling of vGaT-positive puncta (Figure 11A and 11B), thus suggesting that astroglia-derived neurosphere cells transduced with Dlx2 had acquired a GABAergic identity. Finally, electrophysiological recordings demonstrated the GABAergic phenotype of the fate-mapped astroglia expanded as neurospheres and reprogrammed by Dlx2 (*n* = 9; Figure 11C–11E). Consistent with the widespread vGaT expression, Dlx2-expressing neurons received spontaneous synaptic activity displaying slow decay time kinetics characteristic of GABAergic currents (9 out of 10 cells recorded, unpublished data). As shown in Figure 11E, step-depolarisation of a GFP-positive, Dlx2-expressing neuron (Figure 11C and 11D, black asterisk) evoked an autaptic response of the stimulated neuron that was blocked by bicuculline. Four out of 10 Dlx2-transduced neurosphere-derived neurons recorded were found to form GABAergic autapses, despite being analysed at a younger age compared to the adherent astroglia (*n* = 10, age of the cells: 20.1±1.6 DPI; Figure S2A). Consistent with the development of GABAergic networks in Dlx2-transduced cultures, calcium imaging experiments performed at 27 DPI did not reveal any spontaneous Ca^2+^ transients in the analysed DsRed-positive neurons (*n* = 70 neurons recorded, *n* = 2 independent experiments; Figure S5). Thus, culturing astroglia under neurosphere conditions clearly eases the reprogramming towards functional GABAergic neurons by Dlx2 transduction compared to the effects obtained in adherent astroglia (40% versus 6%, neurosphere cells versus adherent astroglia, respectively).

**Figure 12:**
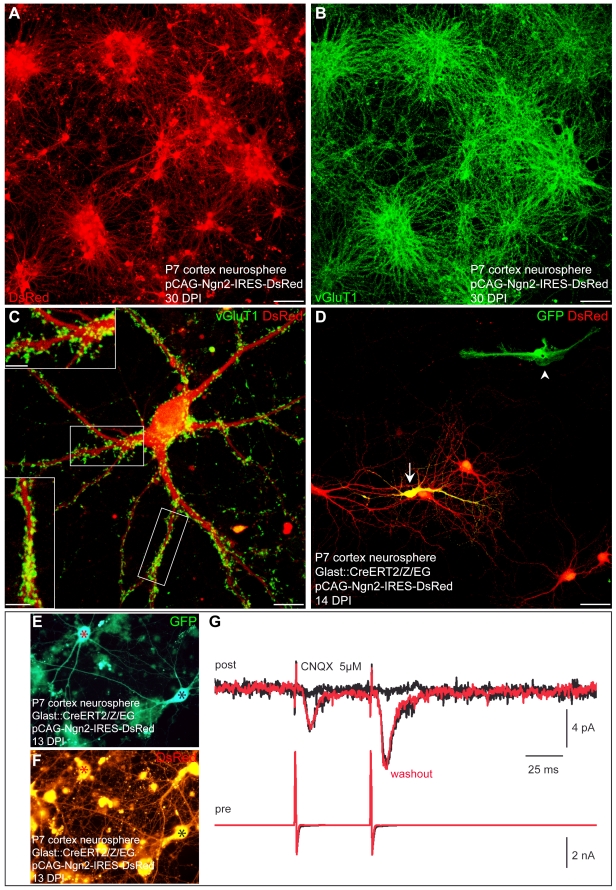
Postnatal cortical astroglia expanded as neurospheres and subsequently reprogrammed by Neurog2 generate synapse-forming glutamatergic neurons. (A) Postnatal cortical astroglia were first expanded as neurospheres and subsequently transduced with a retrovirus encoding Neurog2 and DsRed. Immunostaining for DsRed reveals that virtually all astroglia-derived neurosphere cells differentiate into neurons upon forced expression of Neurog2 at 30 DPI. (B) Micrograph depicting vGluT1 immunocytochemistry of the same culture shown in (A). Note the massive expression of vGluT1-positive puncta indicating that the majority of the Neurog2-expressing neurons acquire a glutamatergic identity. (C) High magnification view of a single Neurog2-reprogrammed cell. Double immunostaining for DsRed (red) and vGluT1 (green) illustrates the dense labelling and the distribution of the vGluT1-positive puncta. Insets show high magnification views of the boxed areas. (D) Fate-mapping analysis using postnatal cortex of tamoxifen induced GLAST::CreERT2/Z/EG mice corroborates the astroglial origin of the cells reprogrammed by Neurog2. The fluorescence micrograph depicts a GFP-positive cell derived from a fate-mapped astroglia and exhibiting a neuronal morphology following transduction with Neurog2 at 14 DPI (arrow). Note the non-reprogrammed GFP-positive astroglial cell in absence of Neurog2 transduction (arrowhead). (E–F) Fluorescence micrographs depicting two fate-mapped GFP-positive neurons (E) co-expressing DsRed at 13 DPI (F), thus indicating reprogramming by Neurog2. The red and the black asterisks mark the presynaptic and the postsynaptic neurons, respectively. (G) Dual recording revealing the glutamatergic nature of the stimulated presynaptic neuron. The presynaptic neuron is step-depolarised in voltage clamp (pair pulse stimulation, lower red trace) evoking inward synaptic currents in a nearby postsynaptic neuron that are completely abolished by CNQX (upper black traces; each trace represents an average of 10 single responses including failures). The upper red trace shows the recovery of the glutamatergic synaptic responses after washout of CNQX, perfectly overlaying the responses prior to CNQX treatment. Scale bars: A, B: 80 µm; C: 11 µm; Insets in C: 5 µm; D: 40 µm.

**Figure 13:**
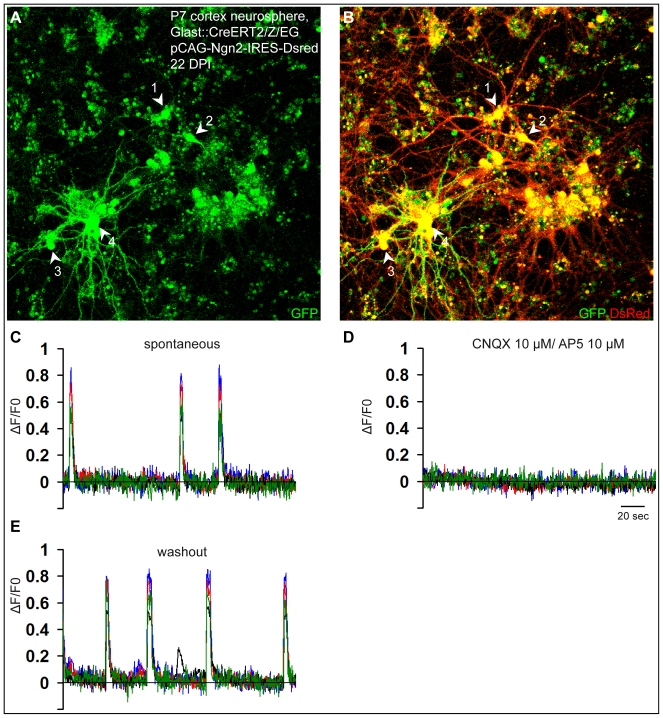
Calcium imaging experiments of fate-mapped postnatal astroglia expanded as neurospheres and subsequently transduced with Neurog2. (A) The fluorescence micrograph depicts GFP-positive neurons derived from fate-mapped astroglia prepared from the postnatal cortex of tamoxifen-induced GLAST::CreERT2/Z/EG mice, that were first expanded as neurospheres and subsequently reprogrammed by forced expression of Neurog2. Arrowheads show the four neurons that are analysed in (C–E). (B) DsRed expression in the same cells shown in (A) indicating neuronal reprogramming by Neurog2. (C–E) The graphs show spontaneous Ca^2+^ transients occurring synchronously in the four recorded neurons shown in (A) and (B), that are totally abolished in the presence of CNQX/AP5 (D, 10 µM each) and re-emerge following washout of the drugs (E). The trace of each individual neuron is shown in a different colour.

Interestingly, astroglia cultured as neurosphere cells could still be reprogrammed by Neurog2 towards a glutamatergic neuronal phenotype. Virtually all astroglia-derived neurosphere cells transduced with Neurog2 differentiated into MAP2-positive neurons (91.4%±2.2%, *n* = 2,272 DsRed-positive cells counted, *n* = 3 independent experiments, in agreement with [7]) that exhibited a large soma size and extended several MAP2-positive processes (Figure S4B and S4D). Again, fate-mapping analysis corroborated the astroglial origin of the reprogrammed cells (Figure 12D–12F). These neurons derived from astroglia-derived neurosphere cells exhibited quite low input resistances similarly to the adherent astroglial cells reprogrammed by Neurog2, therefore suggesting that they had reached a mature neuronal state (Figure S2C; 751.9±118.8 MΩ at 15.0±1.4 DPI (*n* = 10) versus 608.6±125.0 MΩ at 26.8±1.2 DPI (*n* = 14), Neurog2-reprogrammed neurosphere cells versus Neurog2-reprogrammed adherent astroglia, respectively). Immunostaining for vGluT1 revealed a massive labelling of vGluT1-positive puncta indicating that the Neurog2-transduced cells had acquired a glutamatergic identity (Figure 12A–12C). Indeed, electrophysiological pair recordings unambiguously demonstrated the glutamatergic phenotype of the fate-mapped cortical astroglia expanded as neurospheres and reprogrammed by Neurog2 (*n* = 5) (Figure 12E–12G). In addition, step-depolarization of Neurog2-expressing neurons also evoked in several cases a sequence of polysynaptic components consistent with the development of excitatory networks in these cultures (Figure S3G and S3H). Nine out of 21 DsRed-positive neurons recorded exhibited glutamatergic autaptic or synaptic connections (age of the cells: 14.2±0.7 DPI; Figure S2A). In addition, calcium imaging experiments performed 3–4 wk post-infection in these Neurog2-reprogrammed cultures revealed a high degree of self-driven, synchronous excitatory network activity, which was blocked by CNQX and AP5 treatment (97 out of 98 DsRed-positive cells imaged; *n* = 3 independent experiments; Figure 13). As can be appreciated from Figure 13 many of the neurons recruited to the self-driven networks were derived from fate-mapped GFP-positive neurosphere cells indicating their astroglial origin (28 out of 29 fate-mapped GFP/DsRed-double-positive cells imaged).

These data suggest that astroglia initially expanded as neurosphere cells are more plastic in regard to their differentiation into various neuronal subtypes. What may be the molecular changes underlying the increased plasticity of neurosphere cells compared to adherent astroglia given their common astroglial origin? The striking increase in the efficiency of reprogramming by Dlx2 could be accounted for by a loss in the expression of molecular cues associated with a glutamatergic bias. However, quantitative RT-PCR showed that there was no difference in the expression of Emx1 or Emx2 mRNAs [38] between astroglial cells cultured adherently or under neurosphere conditions (Figure 10H and 10I). In contrast, astroglial cells cultured under neurosphere conditions expressed drastically higher levels of Sox2 mRNA compared to adherent astroglia (Figure 10J). Given the high level of expression of Sox2 in neural stem cells [39], these data are in agreement with the observation that culturing postnatal astroglia from the cerebral cortex under neurosphere conditions leads to their de-differentiation towards a more stem cell-like state [33],[34].

### Neurosphere Cells Derived from the Adult Injured Cerebral Cortex Can Be Reprogrammed into Synapse-Forming Functional Neurons

**Figure 14:**
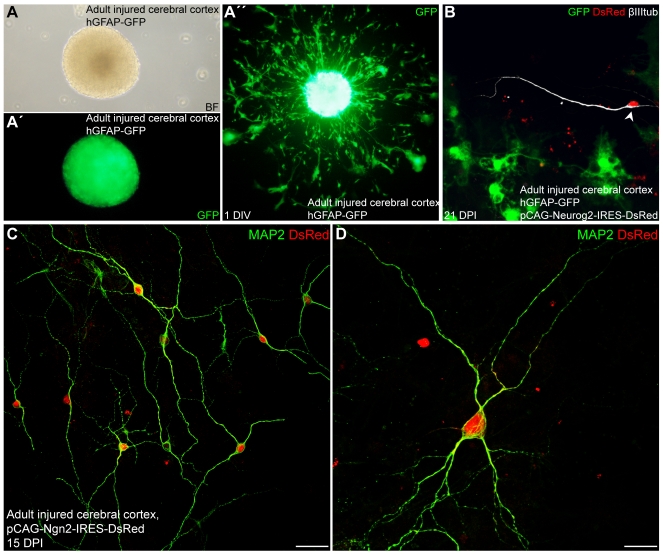
Neurosphere cells derived from reactive astroglia of the injured adult cerebral cortex differentiate into neurons following forced expression of Neurog2. (A–A”) Evidence for the astroglial origin of injury-induced neurospheres. (A) Bright field micrograph of a neurosphere obtained by culturing cells from the stab wound injured cerebral cortex of hGFAP-GFP mice. (A') Live GFP-fluorescence image of the same sphere shown in (A) demonstrates the astroglial nature of the neurosphere cells. (A”) Single plated GFP-positive neurosphere 1 d after plating. (B) Three wk post-infection with Neurog2 encoding retrovirus (DsRed), neurosphere cells from the same sphere shown in (A”) have differentiated into βIII tubulin-positive neurons (white) while losing GFP expression (arrowhead). In contrast, non-transduced astroglia continue expressing high levels of GFP driven from the hGFAP promoter (green). (C) Neurospheres derived from adult injured cortex of wild type mice were plated without dissociation as single neurosphere and subsequently transduced with Neurog2-IRES-DsRed. Double immunostaining for DsRed (red) and MAP2 (green) showing adult cortex-derived neurosphere cells 15 d after transduction with Neurog2 that differentiate into MAP2-positive neurons. (D) The micrograph shows a high magnification of a single MAP2-positive neuron derived from an adult cortex-derived neurosphere cell reprogrammed by Neurog2. Scale bars: C: 40 µm; D: 20 µm.

The above finding that Neurog2 or Dlx2 overexpression can induce with high efficiency the generation of functional neurons from postnatal astroglia-derived neurosphere cells prompted us to examine whether reactive astroglia from the adult cortex following injury can also be reprogrammed to generate functional neurons after prior expansion as neurospheres. Indeed, previous work of our laboratory has shown that following a local injury such as a stab wound lesion, reactive astroglia isolated from the adult cerebral cortex de-differentiate, resume proliferation, and have the capacity to give rise to self-renewing neurospheres in vitro, in contrast to the intact contralateral cortex [16]. To examine the reprogramming potential of reactive astroglia isolated from the adult injured cerebral cortex, we performed local stab wound lesions in the right cortical hemisphere of adult C57BL/6J mice and dissociated both the control and injured cortical hemispheres 3 d later for subsequent neurosphere cultures. While control cortical tissue did not generate neurospheres, the injured hemisphere gave rise to neurospheres as reported [16]. Confirming previous results [16], neurospheres could also be obtained from single GFP reporter-positive cells derived from either GLAST::CreERT2/Z/EG mice (unpublished data) or hGFAP-GFP mice (Figure 14A–14A”). After 1–2 wk, single neurospheres were plated, subsequently transduced with retrovirus encoding Neurog2 and DsRed or Dlx2 and DsRed, and then allowed for differentiation.

When astroglia-derived neurosphere cells obtained from lesioned cortex of wild type mice were transduced with Neurog2, virtually all the DsRed-positive cells had developed into MAP2-positive neurons at 15 DPI (>50 DsRed-positive cells per sphere; 25 spheres analysed, Figure 14C and 14D). Importantly, when GFP-positive neurospheres originating from the injured cortex of hGFAP-GFP mice (Figure 14A–14B) were transduced with Neurog2, we observed the generation of numerous DsRed-positive neurons (total number of neurons >50, 10 spheres analysed). In contrast to untransduced lesion cortex neurosphere cells, GFP expression was lost following neuronal differentiation (Figure 14B), consistent with the astroglia-to-neuron fate change.

**Figure 15:**
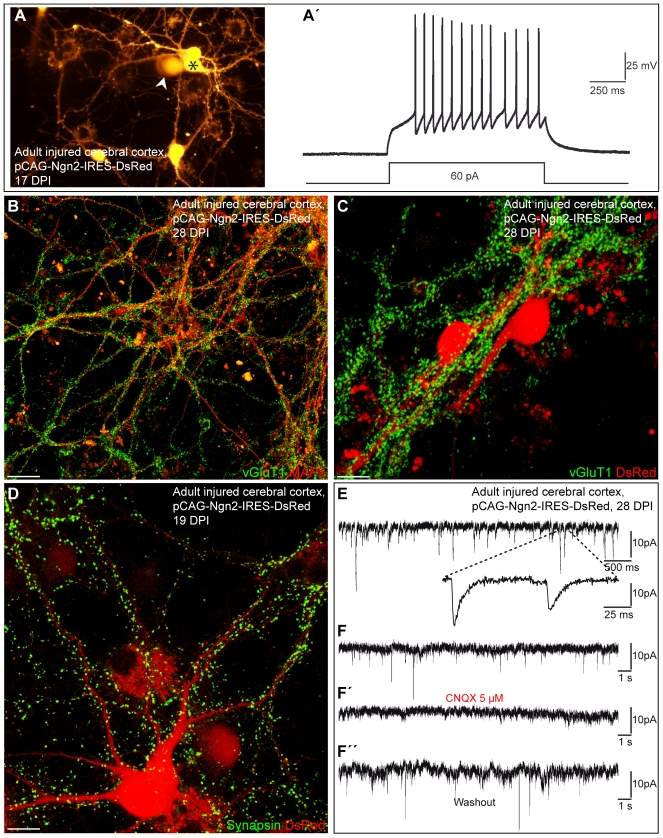
Reactive astroglia from the adult injured cortex expanded as neurospheres generate functional neurons upon forced expression of Neurog2. (A) Fluorescence micrograph depicting a cluster of adult injured cortex-derived neurosphere cells transduced with a retrovirus encoding Neurog2 and DsRed that exhibit a neuronal morphology at 17 DPI. The white arrowhead indicates the tip of the recording pipette filled with DsRed. (A') The graph shows repetitive firing of action potentials in response to step-current injection in the neuron marked by the asterisk in (A). (B) Double immunocytochemistry for MAP2 (red) and vGluT1 (green) shows a dense labelling of vGluT1-positive puncta that outline the MAP2-positive processes emanating from adult cortex-derived neurosphere cells reprogrammed by Neurog2 at 28 DPI. (C) Massive expression of vGluT1-positive puncta (green) outlining the cell body and the processes of two adult cortex-derived neurosphere cells transduced with Neurog2 (red) at 28 DPI. (D) The fluorescence micrograph depicts a Neurog2-expressing neurosphere cell 19 DPI exhibiting a dense punctuate staining for synapsin, indicating the presence of synapses impinging onto this neuron. (E) The graph depicts spontaneous synaptic activity recorded from adult injured cortex-derived neurosphere cells transduced with Neurog2 at 28 DPI. The enlarged trace shows individual synaptic events displaying fast decay time kinetics typical of glutamatergic currents. (F–F”) Spontaneous activity can be blocked by CNQX demonstrating that it is driven by glutamatergic synaptic transmission. Scale bars: B: 25 µm; C, D: 12 µm.

Following step-current injection, Neurog2-expressing neurosphere-derived cells responded with a train of repetitive APs demonstrating their neuronal nature (*n* = 30; Figure 15A and 15A'). In addition, these neurons derived from adult glia exhibited relatively low input resistance values similar to Neurog2-transduced neurons derived from the postnatal cortical neurosphere cells (1,009.2±177.4 MΩ at 21.9±1.2 DPI (*n* = 19) versus 751.9±118.8 MΩ at 15.0±1.4 DPI (*n* = 10), respectively). We next assessed whether these neurons could establish functional synaptic connections. Single adult cortex-derived neurospheres showed a massive immunostaining of vGluT1-positive puncta as shown 28 DPI, that outlined the dense network of intermingled MAP2-positive processes (Figure 15B) and the soma of Neurog2-transduced cells (Figure 15C). Consistent with vGluT1 expression, immunostaining for the presynaptic protein synapsin also revealed a dense labelling of synapsin-positive puncta, thus suggesting the development of synaptic contacts between adult lesioned cortex-derived neurosphere cells (Figure 15D). Furthermore electrophysiological recordings revealed the emergence of CNQX-sensitive spontaneous synaptic currents in these neurons in accordance with vGluT1 and synapsin expression (8 out of 30 cells recorded at 22.5±0.9 DPI; Figure 15E–15F”). These data indicate that adult astroglia-derived neurosphere cells transduced with Neurog2 mature into functional glutamatergic neurons.

To examine the extent of plasticity of glial cells derived from the adult lesioned cortex we also tested as a proof-of-principle experiment whether adult lesioned cortex-derived neurosphere cells could also be directed by forced expression of Dlx2 towards MAP2-expressing neurons (Figure S6A and S6A'). However, Dlx2 reprogrammed neurons were rather few and fragile due to their rather small soma size, thus hampering extensive electrophysiological analysis. Nevertheless we could record from one cell shown in Figure S6, where step-depolarisation evoked an autaptic response that was blocked by bicuculline, indicating the development of functional GABAergic connections (Figure S6B and S6B'). These data show that even adult cells isolated from the injured cortex and expanded as neurospheres can be instructed by forced expression of Neurog2 or Dlx2 to generate mature neurons able to establish functional glutamatergic or GABAergic connections, respectively.

## Discussion

The present study provides four major findings: firstly, it provides new independent experimental evidence based on genetic fate-mapping that astroglia from the postnatal cerebral cortex can be reprogrammed by a single transcription factor into functional neurons; secondly, we have succeeded in overcoming the previous limitations in synaptogenesis of neurons derived from postnatal cortical astroglia by the use of retroviral vectors conveying higher and more persistent levels of neurogenic fate determinants' expression [7]; thirdly, based on the transcription factor used for reprogramming, postnatal astroglia can be directed towards the generation of glutamatergic and GABAergic neurons, providing proof-of-principle evidence that selective subtypes can be generated from the same cells of origin; fourthly, reprogramming efficiency is further enhanced by prior de-differentiation of the astroglia as provided by the expansion under neurosphere conditions. Importantly, using the latter procedure we even succeeded in reprogramming reactive astroglia after adult brain injury. Thus, reprogramming of astroglia towards functional neurons with a single transcription factor is not restricted to postnatal stages but can also be achieved from astroglia of the adult cerebral cortex following injury-induced reactivation.

### Neuronal Reprogramming of Cells Restricted to the Astroglial Lineage

We have previously shown that postnatal astroglia can be reprogrammed into neurons [6],[7]. However, as the astroglial origin of the reprogrammed cells is a very important issue, here we sought to provide new experimental evidence via genetic fate-mapping of astroglia by using the GLAST::CreERT2/Z/EG mouse line developed in our laboratory [23]. To ensure the specificity of this mouse model we showed that virtually all fate-mapped, i.e. GFP-reporter positive, cells remain in the glial lineage under our culture conditions, with the vast majority being identified as astrocytes based on GFAP expression as well as GLAST (unpublished data) and a minor population as oligodendroglial cells, while none of the fate-mapped cells spontaneously gave rise to neurons (Figure 3A and 3A'). These data support the notion that the cells cultured under these conditions do not possess an intrinsic neurogenic potential. This is consistent with the finding of virtually absent endogenous Neurog2 expression compared to cortical precursors isolated at the embryonic stage (Figure 10G) and in agreement with the epigenetic silencing of the neurogenin-1 and -2 loci at the transition between neurogenic and astrogliogenic precursors [40]. These data do not rule out the possibility that in vivo a small subset of astroglial cells can still give rise to neurons as suggested by hGFAP::CreERT2-mediated fate mapping showing that neurons can be generated at early postnatal stages from genetically marked cells [41]. However, so far it could not be experimentally distinguished whether the postnatal generated neurons indeed had been derived from astroglia local to the cerebral cortex or would be derived from astroglial stem cells in the subependymal zone that had subsequently immigrated into the cortex [42]. In any case our cultures do not sustain conditions for the genesis of neurons from astroglia (even in the presence of EGF/FGF2) without forced expression of neurogenic fate determinants.

Yet despite generating only glia when adherently grown in serum containing medium, postnatal astroglia exhibit a remarkable degree of plasticity as indicated by the fact that at least some can give rise to self-renewing, multipotent neurospheres ([36] and present study). The latter fact could be taken as evidence that early postnatal astroglia possess stem cell character, particularly in the light of the notion that cells from the postnatal cerebral cortex may contribute to the pool of radial/astroglial stem cells within the dorsal adult subependymal zone [14] (for review see [43]). However, several lines of evidence argue against a stem cell character of the astroglial cells studied here. Firstly, neither wild-type nor genetically fate-mapped astroglia spontaneously give rise to neurons, which is inconsistent with the stem cell defining hallmark of multipotency (Figure 3A–3B”). Moreover, the large number of neurons generated following forced expression of Neurog2 argues against the possibility that the successfully reprogrammed cells derive from rare stem cells within this culture (Video S1). Secondly, while Dlx2 very efficiently directs adult neural stem cells in vitro towards neurogenesis [26], the responsiveness of adherent astroglia is much more limited, suggesting a reduced susceptibility to Dlx2 transcriptional activity. The third line of evidence is based on the striking difference in Sox2 mRNA levels following expansion of the postnatal astroglia as neurosphere cells. Sox2 is a transcription factor well known to play a key role in neural stem cell self-renewal [39]; thus the massive up-regulation of Sox2 following exposure of astroglia to neurosphere culture conditions suggests that these cells undergo de-differentiation eventually acquiring indeed stem cell properties, while the comparatively lower levels of Sox2 in the adherent cultures would be in agreement with their non-stem cell character. Consistent with the higher degree of plasticity characterizing neural stem cells, the efficiency of reprogramming of astroglia-derived neurosphere cells by Dlx2 was found to reach levels comparable to bona fide neural stem cells (Figure S4; [26]). Intriguingly, exposure of non-stem cell astroglia to EGF/FGF2 in the absence of serum factors may thus mimic similar extrinsic signals encountered by astroglial cells during postnatal development that later give rise to the stem cell compartment in the adult subependymal zone [44]. In fact the conversion of quiescent astrocytes from the adult cerebral cortex into stem cell-like cells following injury ([16] and present study) clearly demonstrates that cells apparently devoid of stem cell properties can acquire stem cell hallmarks such as self-renewal and multipotency when exposed to the appropriate environment.

### Direct Conversion of Postnatal Astroglia into Synapse-Forming Functional Neurons by a Single Transcription Factor

While our previous findings of eliciting neurogenesis by a single transcription factor from postnatal astroglial cells demonstrated the potency of these neurogenic fate determinants [6],[7], a major obstacle towards reprogramming into fully functional neurons was encountered in the failure of the astroglia-derived neurons to provide functional presynaptic output to other neurons [7]. In our previous study neuronal reprogramming was achieved by transcription factor-encoding retroviral vectors that exhibit relatively low levels of overexpression and are subject to substantial silencing in neurons [18]. Of note, long-term expression of an exogenous transcription factor appears to be required for maintaining a new phenotype following cellular reprogramming [45]. For instance, it has been shown that reprogramming of fibroblasts into macrophage-like cells remains unstable, resulting in the loss of macrophage markers, following silencing of retrovirally expressed PU.1 and C/EBPα [46]. Here we demonstrated that stronger and more prolonged expression of neurogenic fate determinants from retroviral constructs more resistant to silencing [20] indeed permits more complete reprogramming of postnatal cortical astroglia towards synapse-forming neurons. In support of our hypothesis, we found that Dlx2 expression driven from a weaker and silencing-prone retroviral vector (pMXIG) [26] resulted in nearly negligible neurogenesis in postnatal astroglia cultures (unpublished data), while the same fate determinant encoded by the pCAG vector induced substantial neurogenesis. Consistent with a more efficient reprogramming via a strong and silencing-resistant retroviral expression system, we found that forced expression of Neurog2 or Dlx2 endowed astroglia-derived neurons not only with the ability to receive synaptic input but also to form functional presynaptic output onto other astroglia-derived neurons to such degree as generating networks of spontaneously active neurons. Thus, one of the major obstacles that may impair incorporation of astroglia-derived neurons into a neuronal network, namely the inability to give rise to functional presynaptic output, can be overcome by appropriate expression of neurogenic fate determinants. Interestingly, neuronal reprogramming of astroglia by Neurog2 towards mature neurons appears to involve similar developmental steps as in newborn neurons during embryonic and adult neurogenesis. For instance, while GABA acts as an inhibitory neurotransmitter onto mature astroglia-derived neurons reprogrammed by Neurog2, at a more immature stage these neurons respond with a rise in free intracellular calcium (Blum et al., submitted), very similar to immature embryonic- or adult-generated neurons [47],[48] and such a differential response may be required for proper maturation to proceed [49].

Of note, despite its continued expression, Neurog2, a transcription factor normally expressed in progenitors and only transiently maintained in postmitotic neurons [50], does not seem to interfere with a surprisingly normal maturation of the reprogrammed cells. Firstly, input resistances of Neurog2-expressing neurons reach levels that correspond well to values observed for neurons recorded in slices from postnatal mice of roughly matching age [51]. Secondly, as a sign of proper morphological maturation dendrites of Neurog2-expressing neurons were covered with spines after 1 mo in culture, in agreement with progressive formation of excitatory synapses. Finally, astroglia-derived neurons reprogrammed by Neurog2 exhibit prominent expression of the Ca^2+^/Calmodulin-dependent kinase IIα subunit, which in vivo is exclusively expressed in excitatory neurons starting from the first postnatal week [22]. Moreover, expression of Ca^2+^/Calmodulin-dependent kinase IIα is a pre-requisite for the occurrence of changes in synaptic efficacy [52], suggesting that Neurog2-reprogrammed cells potentially also acquire the ability to undergo synaptic modification.

The fact that cells committed to the astroglial lineage can be reprogrammed into fully functional synapse-forming neurons also sheds some light on the more general question of whether the conversion across cell lineages induced by a single transcription factor can generate fully differentiated and stable cell fates that closely mirror cell types found in vivo [45]. Our study provides definitive positive evidence that such a cell lineage conversion is indeed possible and does not require passing through a pluripotent ground state. Interestingly, a recent study has shown that even mouse embryonic or perinatal fibroblasts, i.e. cells of the mesodermal lineage, can be converted by combined forced expression of three defined factors (Mash1, Brn2, Myt1l) into functional neurons [53]. Unexpectedly this combination seems to favour the generation of glutamatergic neurons, while the possibility to generate neurons of other phenotypes remains to be explored. Our study indeed reveals for the first time the feasibility of direct conversion of a somatic cell type into distinct neuronal subtypes by selective expression of transcription factors. Moreover, the efficiency of fibroblast conversion into neurons remains markedly lower (∼20%) despite the joint use of three transcription factors. Conversely, we demonstrate here that cells of closer lineage-relationship to ectoderm-derived neurons, namely the astroglia, require only a single transcription factor (Neurog2) to be converted towards fully functional neurons with 60% efficiency. This does not only confirm the notion that lineage reprogramming is achieved with best results by using the closest related cells but is also of profound relevance in regard to the eventual translational potential towards regenerative medicine. Activation of endogenous brain cells towards neuronal repair may be feasible if only one factor needs to be activated, e.g. by transvascular delivery of small molecules and RNAs [54]. In addition, the regional specification of astroglial cells characterized by distinct transcription factor profiles [55],[56] may create a specific bias towards the generation of the type of neurons normally residing within the respective brain region and hence favour the fate conversion into the appropriate neuronal subtypes. Our results therefore further pave the way towards neuronal reprogramming from endogenous cells residing within the brain, circumventing the complications associated with transplantation.

Intriguingly, although the majority of astroglial cells undergoing reprogramming by Neurog2 also undergo cell cycle division, single cell tracking demonstrated that astroglia can give rise to neurons without dividing. These data show that cell division is not a sine qua non condition for successful reprogramming, providing additional evidence for a direct fate conversion by-passing a proliferative state. Future studies will have to reveal whether even adult quiescent astrocytes could be reprogrammed into neurons without the requirement of entering the cell division cycle.

### Subtype Specification of Astroglia-Derived Neurons

Notably, given that expression of neurogenic fate determinants allows for the generation of synapse-forming neurons, we could assess whether distinct transcription factors promote neuronal subtype specification of the reprogrammed cells. Indeed, our immunocytochemical and electrophysiological analysis demonstrated that astroglia derived from the cerebral cortex can be reprogrammed not only towards the generation of glutamatergic neurons by Neurog2, a fate determinant that regulates glutamatergic neuron generation in the developing dorsal telencephalon [8], but also towards the generation of synapse-forming GABAergic neurons by Dlx2. Of note, glutamatergic neurogenesis from cortical astroglia following reprogramming by Neurog2 was accompanied by the (transient) up-regulation of Tbr2 and Tbr1, T-box transcription factors that characterize the genesis of glutamatergic neurons throughout the forebrain [21]. Moreover, Neurog2 induced the expression of the Ca^2+^/Calmodulin-dependent kinase IIα, which is selectively expressed in glutamatergic neurons of the forebrain [22]. The fact that Neurog2-reprogrammed astroglia derived from the cerebral cortex generate glutamatergic neurons rather than other neuronal subtypes which also require Neurog2 expression for their specification, such as spinal cord cholinergic motoneurons, is consistent with the notion that astroglial cells retain region-specific identity characterized by distinct transcription factor profiles [55],[56], in this case dorsal telencephalic cues [57].

In contrast to Neurog2, Dlx2 is normally expressed in progenitor cells derived from the ventral telencephalon and has been shown to play a crucial role in the genesis of GABAergic neurons during development [13] and in adulthood [26] in this region. Yet the fact that astroglial cells of dorsal telencephalic origin can be forced to adopt a “ventral” fate is consistent with previous findings that ventral transcription factors can instruct a GABAergic fate in dorsally derived progenitors including induction of endogenous Dlx gene expression [12],[58]. Of note, however, this cellular competence to respond appropriately to “wrong” regional transcriptional cues is not restricted to relatively unspecified precursors but can be even observed in cells committed to the astroglial lineage.

However, the ability of Dlx2 to induce neuronal reprogramming from cortical astroglia is limited. In contrast to reprogramming induced by Neurog2 that occurred with very high efficiency, only a third of all Dlx2-transduced astroglia differentiated into neurons and most of these exhibited high input resistances typical of immature neurons even after prolonged periods of culturing. Moreover, the apparent impairment of maturation resulted in an even lower number of Dlx2-expressing neurons forming functional GABAergic synapses. Also only a minority of the neurons obtained from Dlx2-reprogrammed astroglia displayed more mature interneuronal firing patterns, with the majority of neurons often responding with one or few spikes to prolonged current injection. Intriguingly, however, among those neurons acquiring mature firing properties we could clearly discern distinct types of patterns which have been classified as regular spiking, stuttering, and low-threshold burst spiking. These data indicate that Dlx2-reprogrammed astroglia can eventually mature into specific interneuron subtypes. Consistent with this we found that a small subset of the reprogrammed cells expressed the calcium binding protein calretinin, which is normally expressed within the cortex in subpopulations of regular or low-threshold burst spiking interneurons [29].

The apparent limitations of Dlx2-mediated astroglia-to-neuron fate conversion could be due to inaccessibility of some downstream targets of Dlx2 in cortex-derived astroglia or to an overall lower potency of Dlx2 compared to Neurog2 in reprogramming and/or the need for additional co-factors missing in cortical astroglia. Along these lines we examined whether co-expression of Mash1 with Dlx2 may further promote the maturation and specification of astroglia into synapse-forming interneurons. Mash1 is a ventral telencephalic transcription factor upstream of Dlx2 in the cascade of interneuron specification during development [11],[12]. Importantly, while being upstream of Dlx2, which is a direct transcriptional target of Mash1 [30], the latter factor is also known to activate targets that are not shared with Dlx2, suggesting that complete interneuron specification may require the activity of both factors [31],[32]. In agreement with this, we found that co-expression of Mash1 and Dlx2 promoted neurogenesis from astroglia in a synergistic manner to levels similar to Neurog2 (Figure 9). Moreover, input resistances were lower compared to those of Dlx2 only expressing neurons. Finally, the relative proportion of neurons exhibiting more mature interneuronal firing patterns was increased (Figure 8). However, there was no apparent enhancement of synapse formation following co-expression. This may point to the possibility that full interneuronal maturation requires extrinsic signals provided by their target cells, i.e. excitatory neurons, which are absent in cultures from Dlx2-reprogrammed astroglia. Alternatively, additional transcription factors such as Dlx5/Dlx6 may be required for complete maturation to occur.

Given that different subtypes of GABAergic neurons are generated in the telencephalon ranging from medium spiny projection neurons of the striatum to various types of aspiny interneurons throughout the telencephalon [29], it will be of great interest to develop strategies to further refine the subtype specification of GABAergic neurons generated upon forced Dlx2 or Mash1/Dlx2 expression, by using additional transcriptional cues in order to generate the full spectrum of GABAergic interneurons. Such directing of astroglia towards specific interneuron phenotypes may allow for the development of alternative approaches for the treatment of pharmaco-resistant epileptic disorders, particularly during early childhood [59],[60].

Interestingly, culturing astroglial cells prior to transduction under neurosphere conditions improved the efficiency of reprogramming towards GABAergic neurons quite dramatically. Indeed, the synapse-forming capacity of Dlx2-expressing astroglia-derived neurosphere cells exceeded that of adherent astroglia-derived neurons expressing Dlx2 by a factor of seven. In addition, neurons derived from Dlx2-expressing neurosphere cells exhibited significantly lower input resistances consistent with a more mature state.

These findings are not only of great importance in regard to eliciting the generation of neurons of different subtypes but may also support the concept that culturing cells under neurosphere conditions (in serum-free medium containing high levels of EGF and FGF2) induces the erasure of region-specific transcription [33],[34]. Indeed culturing neural cells of different origins under neurosphere conditions has been shown to induce a partial loss of region-specific transcription factor expression while resulting in the up-regulation of Olig2 and Mash1 [34], providing a more permissive transcriptional environment that favours reprogramming towards distinct neuronal subtypes. However, we found no striking differences in the expression of the mRNAs encoding the cortical patterning factors Emx1 and Emx2 between cortical astroglia cultured adherently or expanded under neurosphere condition (Figure 10H and 10I). In contrast, the two populations differed drastically in their expression levels of Sox2 mRNA (Figure 10J), which may indicate a more stem cell-like status of the astroglia under neurosphere conditions and which may be the molecular correlate for their higher degree of plasticity.

### Reprogramming of Adult Injured Cortex-Derived Neurosphere Cells into Functional Synapse-Forming Neurons

The successful reprogramming of astroglia following expansion under neurosphere conditions also encouraged us to investigate whether neurosphere cells derived from the adult cerebral cortex after injury can be similarly directed towards neurogenesis. Thus, we assessed here whether adult cortex-derived neurosphere cells also can be driven towards fully functional neurons following forced expression of Neurog2 or Dlx2. Indeed, virtually all the cells expressing Neurog2 acquired a neuronal identity as revealed by MAP2 staining and their ability to fire APs. Moreover, we also provide evidence by vGluT1 immunoreactivity that the neurons derived from these lesion cortex-derived neurosphere cells undergo a subtype specification similar to reprogrammed postnatal astroglia and differentiate into glutamatergic neurons. Consistent with the development of functional synapses by Neurog2-reprogrammed adult cortex-derived neurosphere cells, we could record spontaneous glutamatergic events in these cultures. Conversely, following forced expression of Dlx2, we could observe the generation of functional GABAergic neurons from adult cortex-derived neurosphere cells, indicating that the same dichotomy of subtype specification observed in postnatal astroglia also holds true for adult cortex-derived neurosphere cells following injury. While this first demonstration that functional neurons of different subtypes even undergoing synaptic connectivity can be derived in vitro from adult glial cells isolated from the injured cortex is an exciting step forward to utilize endogenous glial cells for repair of neurons [61]–[63], it will still be a major challenge to translate these in vitro findings into the context of the injured brain.

## Materials and Methods

### Animals and Tamoxifen Treatment

Experiments were conducted either on C57BL/6J mice or transgenic GLAST::CreERT2/Z/EG double heterozygous mice. Briefly, heterozygous GLAST::CreERT2 mice [23] were crossed with the Z/EG reporter mouse line [24] to generate double heterozygous mutants. The activation of the tamoxifen-inducible form of the Cre recombinase was done as follows: From postnatal day 2, i.e. at the peak of cortical astrogliogenesis [64], until sacrifice at postnatal day 7, tamoxifen (20 mg/mL, dissolved in corn oil, Sigma-Aldrich, Munich, Germany) was administered as described by Mori et al. [23] to mothers and mice pups thus received tamoxifen via the milk from their mother during lactation. Using the same mouse line as in this study, Mori et al. found that by recombination already at E18 virtually all fate-mapped cells give rise to glia [23], indicating that at that stage GLAST expressing cells have lost their intrinsic neurogenic potential.

In some experiments, hGFAP-GFP transgenic mice were also used [65]. All animal procedures were carried out in accordance with the policies of the use of Animals and Humans in Neuroscience Research, revised and approved by the Society of Neuroscience and the state of Bavaria under licence number 55.2-1-54-2531-144/07. All efforts were made to minimize animal suffering and to reduce the number of animals used.

### Cell Cultures of Astroglia from the Postnatal Cerebral Cortex

#### Primary cultures from postnatal cortical astroglia

For culturing postnatal astroglia we followed the procedure described previously by Heins et al. (2002) [6]. After removal of the meninges, grey matter tissue from P5–P7 cerebral cortex of C57BL/6J or GLAST::CreERT2/Z/EG mice was dissected and dissociated mechanically. Subsequently, cells were centrifuged for 5 min at 1,000 rpm, re-suspended, and plated in a medium consisting of DMEM/F12 (Gibco), 3.5 mM glucose (Sigma), 10% fetal calf serum (Gibco), 5% horse serum (Gibco), penicillin/streptomycin (Gibco), and supplemented with B27 (Gibco), 10 ng/mL epidermal growth factor (EGF, Roche), and 10 ng/mL fibroblast growth factor 2 (FGF2, Roche). Contaminating oligodendrocyte precursor cells were removed by brusquely shaking the culture flasks several times. Cells were passaged after 1 wk using trypsin/EDTA (Gibco) and plated on poly-D-lysine (Sigma-Aldrich, Munich, Germany) glass coated coverslips at a density of 60,000 cells per coverslip (in 24-well plates; BD Biosciences, Erembodegem, Belgium) in the same medium. The vast majority of the cells (>90%) in these cultures were positive for glial fibrillary acidic protein (GFAP) as previously described [7].

#### Neurosphere cultures from postnatal cortical astroglia

Early postnatal astroglia have been shown to give rise to neurospheres until postnatal day 11 [36]. For culturing neurosphere cells from postnatal cerebral cortex we followed the protocol described by Johansson et al. (1999) [66]. After removal of the meninges, grey matter tissue from P5–P7 cerebral cortex of C57BL/6J or GLAST::CreERT2/Z/EG mice was enzymatically dissociated in 0.7 mg/mL hyaluronic acid (Sigma-Aldrich, Munich, Germany) and 1.33 mg/mL trypsin (Sigma-Aldrich) in Hanks' Balanced Salt Solution (HBSS, Invitrogen, Karlsruhe, Germany) with 2 mM glucose and buffered with HEPES (Invitrogen) at 37°C for 30 min. Trypsinization was stopped by adding an equal volume of an ice cold medium consisting of 4% bovine serum albumine (BSA, Sigma-Aldrich) in Earle's Balanced Salt Solution (EBSS, Invitrogen), buffered with 20 mM HEPES (Invitrogen). The cells were then centrifuged at 1,300 rpm for 5 min, re-suspended in ice cold medium consisting of 0.9 M sucrose (Sigma-Aldrich) in 0.5× HBSS, and centrifuged for 10 min at 2,000 rpm. The cell pellet was re-suspended in 2 mL ice cold medium consisting of 4% BSA in EBSS buffered with 20 mM HEPES, and the cell suspension was placed on top of 12 mL of the same medium and centrifuged for 7 min at 1,500 rpm. The cell pellet was finally re-suspended in Dulbecco's Modified Eagle Medium/F12 (DMEM/F12, Invitrogen) supplemented with B27 (Invitrogen), 2 mM glutamine (Sigma-Aldrich), 100 units/mL penicillin (Invitrogen), 100 µg/mL streptomycin (Invitrogen), buffered with 8 mM HEPES, and the cells were placed in uncoated tissue culture flasks (BD Biosciences, Erembodegem, Belgium) at a density of 5,000 cells/mL. To induce neurosphere formation, 20 ng/mL epidermal growth factor (EGF; Chemicon, Eagle Close, United Kingdom) and 10 ng/mL fibroblast growth factor 2 (FGF2; Invitrogen) were added to the culture medium at the beginning and every other day. After 5–7 d in culture, neurosphere cells were enzymatically dissociated with trypsin (Invitrogen) and plated on poly-D-lysine (Sigma-Aldrich) coated coverslips at a density of 10×10^4^ cells per coverslip (in 24-well plates, BD Biosciences) in a medium consisting of DMEM/F12 supplemented with B27, EGF, FGF2, penicillin/streptomycin, and buffered with HEPES (i.e. neurosphere medium).

In some experiments, tissue from P5–P7 cerebral cortex from hGFAP-GFP mice was dissected as described above. Cells were serially diluted and plated as single cell in Terasaki plates (60 wells) in 20 µL of neurosphere medium per well. Immediately after plating, wells were carefully analyzed using an inverted epifluorescence microscope. Wells containing only one single GFP-positive cell were selected for further analysis, whereas wells containing either GFP-negative cells or more than one cell were discarded. EGF and FGF2 were added every other day to the selected wells and neurosphere formation from the selected GFP-positive cells was followed over 1 wk. Single GFP-positive neurospheres were collected, dissociated, and neurosphere cells were plated on poly-D-lysine coated coverslips as described above.

### Retroviral Transduction

Retroviral transduction of astroglia cultured as adherent astroglia or expanded as neurosphere cells was performed 2–3 h after plating on coverslips, using VSV-G (vesicular stomatitis virus glycoprotein)-pseudotyped retroviruses encoding neurogenic fate determinants. Neurog2 or Dlx2 were expressed under control of an internal chicken β-actin promoter with cytomegalovirus enhancer (pCAG) together with DsRed located behind an internal ribosomal entry site (IRES) [20]. The Neurog2 coding cDNA was subcloned from the pCLIG-Neurog2 construct [67] into the EcoRI site of the pSKSP shuttle vector, from where it was then subcloned between the 5′SfiI and 3′PmeI restriction sites of the pCAG retroviral vector to generate pCAG-Neurog2-IRES-DsRed. The Dlx2 coding cDNA was subcloned from the pMXIG-Dlx2 construct [26] and inserted into the pCAG retroviral vector following the same cloning strategy to generate pCAG-Dlx2-IRES-DsRed. For control, cultures were transduced with a virus encoding only DsRed behind an IRES under control of the chicken β-actin promoter (pCAG-IRES-DsRed). Viral particles were produced using gpg helperfree packaging cells to generate VSV-G (vesicular stomatitis virus glycoprotein)-pseudotyped viral particles [68]. Viral particles were titered by clonal analysis after transduction of E14 cortical cultures.

Twenty-four hours after transduction, the medium of astroglia cultured as adherent astroglia was completely replaced by a differentiation medium consisting of DMEM/F12, 3.5 mM glucose, penicillin/streptomycin, and B27 supplement, and the cells were allowed for differentiation for different time periods. Similarly, the medium of astroglia-derived neurosphere cells was replaced by a differentiation medium consisting of DMEM/F12, 3.5 mM glucose, penicillin/streptomycin, supplemented with B27, and buffered with HEPES. As it has been found that Brain-Derived Neurotrophic Factor (BDNF) is required for robust synapse formation of neurons derived from neural stem cells [69], 20 ng/mL of BDNF (Calbiochem) were added to the cultures every fourth day during the differentiation period. Cells were cultured at a CO2 concentration of 9%, resulting in a pH of the differentiation medium of ∼7.2. After 7–41 d following transduction, cells were used either for immunocytochemistry, electrophysiology, or calcium imaging experiments. The number of days after retroviral transduction is indicated as DPI.

### Astroglia Transfection

Transfection via DNA-liposome complexes was performed 2 h after plating of passaged cortical astroglia on poly-D-lysine coated 24-well tissue plates. DNA-liposome complexes were prepared in Optimem medium (Invitrogen) using the retroviral plasmid pCAG-Neurog2-IRES-DsRed or the control plasmid pCAG-IRES-DsRed and Lipofectamine 2000 (Invitrogen) as cationic liposome formulation. Astrocyte cultures were exposed to DNA-liposome complexes at a concentration of 0.5 µg DNA per 400 µL of Optimem medium for 4 h. Subsequently the medium was replaced by differentiation medium consisting of DMEM/F12, 3.5 mM glucose, penicillin/streptomycin, and B27 supplement, and the 24-well tissue plates were placed into the time-lapse incubating chamber.

### Time-Lapse Video Microscopy

Time-lapse video microscopy [70],[71] of P7 cortical astrocyte cultures was performed with a cell observer (Zeiss) at a constant temperature of 37°C and 8% CO_2_. Phase contrast images were acquired every 4 min and fluorescence images every 6–12 h for 6–8 d using a 20× phase contrast objective (Zeiss) and an AxioCamHRm camera with a self-written VBA module remote controlling Zeiss AxioVision 4.7 software [72]. Single-cell tracking was performed using a self-written computer program (TTT) [72]. Videos were assembled using Image J 1.42q (National Institute of Health, USA) software and are played at speed of 4 frames per second.

### Surgery of Adult Animals

Adult C57BL/6J mice of 8–10 wk of age (20–25 g) were injured in the neocortex as described previously [61]. Briefly, mice received Rimadyl (4 mg/kg, s.c., Carprofen) as analgesic treatment and were anesthetized with ketamine (100 mg/kg, i.p., Ketavet, GE Healthcare, Germany) and xylazine (5 mg/kg, i.p., Rompun, Bayer, Germany) and placed in a stereotaxic frame in a flat skull position. After trepanation, a stab wound was made in the right cerebral sensorimotor cortex by using a sharp and thin scalpel (Ophthalmic Corneal V-lance knife, Alcon, Germany) at the following coordinates: anteroposterior (AP)  = from −1.6 to −2.4, mediolateral (ML)  = −1.5, dorsoventral (DV)  = −0.6 mm with Bregma as reference. After surgery, mice were housed in individual Plexiglas cages with food and water *ad libitum* and kept in a 12 h light-dark cycle (room temperature  = 22±1°C).

### Isolation of Cortical Cells from Adult Mice and Neurosphere Cultures

Three days after stab wound lesion, mice were killed by cervical dislocation following euthanasia in rising CO2 concentrations. After removal of the meninges grey matter tissue surrounding the stab wound injury of the neocortex and a similar piece at the same rostrocaudal level in the contralateral hemisphere were dissected and neurospheres were generated as described above.

After 7–14 d, neurospheres generated from the adult injured cerebral cortex were collected and plated as single neurosphere on poly-D-lysine (Sigma-Aldrich) coated coverslips in 24-well plates (BD Biosciences) in a medium consisting of DMEM/F12 supplemented with B27, EGF, FGF2, penicillin/streptomycin, and buffered with HEPES. Two to 3 h after plating on coverslips, neurosphere cells were transduced as described above with retroviral vectors encoding Neurog2 (pCAG-Neurog2-IRES-DsRed), Dlx2 (pCAG-Dlx2-IRES-DsRed), or the control retrovirus (pCAG-IRES-DsRed) and were then allowed for differentiation.

### Immunocytochemistry

For immunocytochemistry, cultures were fixed in 4% paraformaldehyde (PFA) in phosphate buffered saline (PBS) for 15 min at room temperature. Cells were first pretreated in 0.5% Triton X-100 in PBS for 30 min, followed by incubation in 2% BSA and 0.5% Triton X-100 in PBS for 30 min. Primary antibodies were incubated on specimen overnight at 4°C in 2% BSA, 0.5% Triton X-100 in PBS. The following primary antibodies were used: anti-GFP (GFP, chicken, 1∶1000, Aves Labs, *GFP-1020*), polyclonal anti-Glial Fibrillary Acidic Protein (GFAP, rabbit, 1∶4000, DakoCytomation, *Z0334*), polyclonal anti-Red Fluorescent Protein (RFP, rabbit, 1∶500, Chemicon, *AB3216*), polyclonal anti-RFP (rabbit, Rockland, 1∶2000, *600-401-379*), polyclonal anti-vesicular glutamate transporter 1 (vGluT11, rabbit, 1∶1000, Synaptic Systems, *135302*), monoclonal anti-Microtubule Associated Protein 2 (MAP2, mouse IgG1, 1∶200, Sigma-Aldrich, *M4403*), monoclonal anti-synapsin 1 (mouse IgG2, 1∶2000, Synaptic Systems, *106001*), polyclonal anti-vGaT (guinea pig, 1∶200, Synaptic Systems, *131004*), polyclonal anti-Tbr1 (rabbit, 1∶1000, Millipore, AB9616), polyclonal anti-Tbr2 (rabbit, 1∶500, Millipore, AB9618), monoclonal anti-βIII tubulin (mouse IgG2b, 1∶500, Sigma, T8660), polyclonal anti-GAD1(GAD67) (rabbit, 1∶500, Synaptic Systems, 198013), monoclonal anti-calretinin (mouse IgG1, 1∶200, Millipore, MAB1568), and monoclonal anti-CaM kinase IIα (mouse IgG1, 1∶200, Abcam, ab2725). After extensive washing in PBS, cells were incubated with appropriate species- or subclass-specific secondary antibodies conjugated to Cy™2, Cy™3, Cy™5 (1∶500, Jackson ImmunoResearch), Alexa Fluor 488 (1∶500, Invitrogen), FITC (fluorescein isothiocyanate, 1∶500, Jackson ImmunoResearch), TRITC (tetramethyl rhodamine isothiocyanate, 1∶500, Jackson ImmunoResearch), or biotin (1∶500, Jackson ImmunoResearch or Vector Laboratories) for 2 h in the dark at room temperature, followed by extensive washing in PBS. Following treatment with secondary antibodies conjugated to biotin, cells were subsequently incubated for 2 h at room temperature with AMCA streptavidin (1∶200, Vector Laboratories) or Alexa Fluor 647 streptavidin (1∶500, Invitrogen). Coverslips were finally mounted onto a glass slide with an anti-fading mounting medium (Aqua Poly/Mount; Polysciences, Warrington, PA).

### Microscopy Analysis

Stainings were first examined with an epifluorescence microscope (BX61, Olympus, Hamburg, Germany) equipped with the appropriate filter sets. Stainings were further analyzed with laser-scanning confocal microscopes (SP5, Leica, Wetzlar, Germany or LSM710, Carl Zeiss, Göttingen, Germany). Z-stacks of digital images were captured using the LAS AF (Leica) or ZEN software (Carl Zeiss). Single confocal images were then extracted from the Z-stacks. Alternatively, the Z-stacks were collapsed in one resulting picture using the maximum intensity projection function provided by the above mentioned softwares.

### Cell Counts and Statistical Analysis

Cell counts were performed by taking pictures of several randomly selected views per coverslip analysed by means of a Zeiss LSM 710 confocal microscope using a 25× objective. Subsequently, pictures were analysed for cell quantification using Image J 1.42q (National Institute of Health, USA) software. For each quantification, values are given as mean ± SEM. Cell counting data from reprogramming induced by Dlx2, Mash2, and Mash1 in combination with Dlx2 were subjected to a two-tailed Student's *t* test for statistical significance. Differences were considered statistically significant when the probability value was <0.05.

### Electrophysiology

Electrophysiological properties of neurons derived from reprogrammed astroglial cells were analyzed 11–41 d following retroviral transduction. Single or dual perforated patch-clamp recordings [73],[74] were performed at room temperature with amphotericin-B (Calbiochem) for perforation. Micropipettes were made from borosilicate glass capillaries (Garner, Claremont, CA, USA). Pipettes were tip-filled with internal solution and back-filled with internal solution containing 200 µg/mL amphotericin-B. The electrodes had resistances of 2–2.5 MΩ. The internal solution contained 136.5 mM K-gluconate, 17.5 mM KCl, 9 mM NaCl, 1 mM MgCl_2_, 10 mM HEPES, and 0.2 mM EGTA (pH 7.4) at an osmolarity of 300 mOsm. The external solution contained 150 mM NaCl, 3 mM KCl, 3 mM CaCl_2_, 2 mM MgCl_2_, 10 mM HEPES, and 5 mM glucose (pH 7.4) at an osmolarity of 310 mOsm. The recording chamber was continuously perfused with external solution at a rate of 0.5 mL/min.

Cells were visualized with an epifluorescence microscope (Axioskop2, Carl Zeiss) equipped with the appropriate filter sets. For patch clamp recordings, virally transduced cells were selected on the basis of their DsRed immunoreactivity. In addition, to ascertain the astroglial origin of the recorded neurons, DsRed- and GFP-expressing cells from GLAST::CreERT2/Z/EG animals were also selected for patch clamp recordings. Digital pictures of the recorded cells were acquired using a digital camera (AxioCam, Carl Zeiss).

Signals were sampled at 10 kHz with Axopatch 200B patch-clamp amplifiers (Axon Instruments, Foster City, CA, USA), filtered at 5 kHz and analyzed with Clampfit 9.2 software (Axon Instruments). For assessing a cell's ability to fire APs, cells received depolarizing step-current injections. AP amplitudes were measured by subtracting the threshold voltage of the AP from the AP maximum amplitude. For determining input resistance, hyperpolarizing currents of small amplitudes were injected into the cells under current clamp condition at a holding potential of −70 mV and input resistances were calculated from the corresponding voltage deviation.

To examine spontaneous synaptic input into a given neuron, cells were kept in voltage clamp at a holding potential of −70 mV and synaptic events were recorded throughout a period of 1 to 5 min. In order to assess autaptic connections, single cells were step-depolarized in voltage clamp for 1 ms from −70 to +30 mV at a frequency of 0.05 Hz and responses were recorded in the same cell. Responses were considered to be autaptic when they occurred within 3 ms after the step-depolarization [75]. Synaptic connectivity was investigated by means of pair recordings in voltage clamp mode. One neuron was stimulated at low frequency (0.05–0.1 Hz) by a 1 ms step-depolarization from −70 to +30 mV and the response was recorded from the other neuron, and vice versa. To determine the nature of the autaptic or synaptic responses, neurons were step-depolarized as described above and we assessed whether responses could be abolished in the presence of either the GABA_A_ receptor antagonist bicuculline (10 µM) or the AMPA/kainate receptor antagonist CNQX (5 µM). Finally, the recovery of the autaptic or synaptic response was assessed following washout of the pharmacological drugs.

### Confocal Calcium Imaging

Neurons derived from reprogrammed astroglial cells were further analyzed 22, 27, and 29 d after retroviral transduction with calcium imaging experiments. A 5 mM stock solution of Oregon-Green BAPTA1, AM (K_D_: 170 nM, Invitrogen, *O6807*) was prepared in 8.9 µL 20% Pluronic F-127 (Invitrogen) in dimethylsulfoxide (DMSO) by means of a sonifier bath (Bandelin, Berlin, Germany) for 3 min. Reprogrammed astroglial cells were incubated with 5 µM Oregon-BAPTA1, AM in artificial cerebrospinal fluid solution (ACSF: in mM: 127 NaCl, 4.5 KCl, 2.5 NaH_2_PO_4_, 2 CaCl_2_, 2 MgCl_2_, 23 NaHCO_3_, and 25 D-glucose., bubbled with 95% O_2_/5% CO_2_.). The incubation of neurons with the dye-ACSF was performed in a cell culture incubator (37°C, 9% CO_2_) for a loading time of 10–15 min. Cells were washed with ACSF in a perfusion chamber (Volume: 150–200 µL) for at least 10–20 min with a flow rate of approximately 2–3 mL/min. Cells were imaged under continuous perfusion with ACSF solution at 26–30°C. Ligands (Ascent scientific) were bath-applied and used at the following concentrations: CNQX (10 µM), D-AP5 (10 µM), and tetrodotoxin (TTX, 500 nM). For confocal Ca^2+^ imaging (256×256 pixels, 2.16 Hz), an inverted confocal microscope (Olympus IX70, equipped with a Fluoview 300 laser scanning system) was used in combination with an Olympus, UPlanApo 20×/0.7 objective. Transduced cells were identified by means of DsRed expression. Oregon Green-derived fluorescence was excited with a 488 nm laser line (emission filter: band pass 510/540 nm). DsRed was excited at 543 nm (emission filter: band pass: 580 nm±40 nm).

In some experiments, astroglia from GLAST::CreERT2/Z/EG mice, reprogrammed by forced expression of neurogenic fate determinants, were used for calcium imaging experiments. To ascertain the astroglial origin of the DsRed-positive reprogrammed cells that will be analyzed, GFP expression was first pictured at the confocal microscope and was carefully bleached by using the 488-laser afterwards. The DsRed-positive reprogrammed astroglial cells were subsequently incubated with Oregon Green and calcium imaging experiments were processed as described above.

Images were analyzed using IMAGEJ software (WS Rasband, IMAGEJ, US National Institutes of Health, Bethesda, Maryland, USA, http://rsb.info.nih.gov/ij/, 1997–2006). XY-time Calcium imaging results were analyzed by a region-of-interest analysis (pixel intensity) in the extended TIFF format as described before [76].

### Quantitative RT-PCR

Total RNA was extracted with RNeasy Plus MicroKit (Qiagen), according to the manufacturer's instructions. 1–1.5 µg of total RNA was retro-transcribed using Super-ScriptIII Reverse Transcriptase (Invitrogen) and random primers. Each cDNA was diluted one to ten, and 2 µl was used for each real-time reaction. mRNA quantitation was performed on a DNA Engine Opticon 2 System (Bio-Rad) following the manufacturer's protocol using the IQ SYBR Green SuperMix (Bio-Rad). The following oligonucleotide primers were used for the qPCR: *Gapdh*, *Emx1*, and *Emx2* [34]; *Sox2* [77], *Ngn2* [78], *βIIItub* [79], *Glt1* [80], *Glu1* (Glutamine Synthetase) (CCTGGACCCCAAGGCCCGTA; TGGCAGCCTGCACCATTCCAG), *Aldh1l1* (TGTTTGGCCAGGAGGTTTAC; AGGTCACCAGTGTCCAGACC), *S100β* (GATGTCTTCCACCAGTACTCC; CTCATGTTCAAAGAACTCAT), and *Gfap* [81]. The amount of each gene was analyzed in triplicate, and the analysis was repeated on at least three independent samples (*n = *3 for NPC and postnatal neurospheres, *n = *5 for postnatal adherent astrocytes). Data analysis was performed with the ΔΔCt method [82].

## Supporting Information

Figure S1 **Neuronal reprogramming of astroglia involves down-regulation of GFAP and up-regulation of Tbr2 and Tbr1.** (A–A') Down-regulation of GFAP in astroglia-derived neurons. Astroglia culture 6 d post-transduction with Neurog2-IRES-DsRed encoding retrovirus. (A) Note that with the exception of the two cells labelled by the arrowheads depicting DAPI positive nuclei (blue) devoid of any GFAP immunoreactivity, all other cells labelled by nuclear DAPI staining are GFAP-positive (green). (A') The two GFAP-negative cells are DsRed-positive (indicating Neurog2 expression) and are βIII tubulin-positive (white) revealing their neuronal identity (arrowheads). (B–B') Expression of Tbr2 in astroglia undergoing reprogramming at 4 DPI. (B) Immunostaining for DsRed (red) and βIII tubulin (green) shows five cells transduced with Neurog2-IRES-DsRed encoding retrovirus, one of which already developed into a neuron, while in the other cells βIII tubulin expression has barely commenced. (B') Immunostaining for Tbr2 (white, arrowheads) reveals expression in those transduced astroglia which still exhibit glia-like morphology (four out of five cells). This is consistent with the early expression of Tbr2 in the cascade of transcription factors steering glutamatergic neurogenesis [21]. (C–C') Expression of Tbr1 in neurons derived from Neurog2-transduced astroglia at 7 DPI. (C) Immunostaining for DsRed (red) and βIII tubulin (green) reveals five reprogrammed cells. (C') Immunostaining for Tbr1 (white, arrowheads) reveals the up-regulation of Tbr1 by Neurog2 in a large subset (four out of five cells) of Neurog2-transduced cells as reported previously [7].
(7.08 MB TIF)

Figure S2 **Type of synaptic responses and input resistances of postnatal astroglia-derived neurons reprogrammed by Neurog2 or Dlx2.** (A) Summary of the type of autaptic/synaptic responses that could be evoked from astroglia reprogrammed by forced expression of Neurog2, Dlx2, or Mash1 in combination with Dlx2, following culturing under adherent (Astro) or neurosphere conditions (NS). The number of neurons exhibiting autaptic/synaptic connections is expressed as percentage of recorded neurons. Note that astroglia-derived neurons reprogrammed by Neurog2 or Dlx2 in adherent astroglia cultures were recorded at 24.6±0.9 DPI or 26.9±1.4 DPI, respectively. Astroglia-derived neurosphere cells transduced with Neurog2 or Dlx2 were recorded at 14.2±0.7 DPI or 20.1±1.6 DPI, respectively. (B) The graph plots input resistance values over time (MΩ) of astroglia-derived neurons reprogrammed by Dlx2, following culturing under adherent (triangles) or neurosphere conditions (squares), respectively. GFP reporter-positive fate-mapped cells are depicted with green colour. (C) The graph plots input resistance values over time (MΩ) of astroglia-derived neurons reprogrammed by Neurog2, following culturing under adherent (triangles) or neurosphere conditions (squares), respectively. GFP reporter-positive fate-mapped cells are depicted with green colour.
(0.45 MB TIF)

Figure S3 **Postnatal astroglia isolated from hGFAP-GFP mice and expanded as neurospheres generate glutamatergic neurons following forced expression of Neurog2.** (A) To ascertain the astroglial origin of the neurosphere founder cells, cortical tissue from P5–P7 hGFAP-GFP mice was cultured as single cell in Terasaki plates. (B) Fluorescence micrograph depicting cell division of a singly plated GFP-positive astroglial cell. (C–D) The majority of singly plated GFP-positive astroglia gives rise to GFP-positive neurospheres (C) that increase in size over time in culture (D). (E–F) Double immunostaining for DsRed (red) and GFP (green) of astroglia-derived neurospheres as shown in (D), 17 d after neurosphere dissociation and subsequent transduction with Neurog2-IRES-DsRed. (E) Example of a non-transduced cell (i.e., DsRed-negative) expressing GFP and exhibiting a glial morphology. (F) In contrast, following transduction with Neurog2-DsRed, neurosphere cells lose GFP expression and differentiate into neurons. (G) The fluorescence micrograph depicts a cluster of Neurog2-expressing neurosphere cells, derived from singly plated GFP-positive astroglia that lost GFP expression and exhibit a neuronal morphology 17 DPI. (H) The graph shows an evoked sequence of both autaptic and polysynaptic events following step-depolarisation of the neuron marked by the asterisk in (G), suggesting that Neurog2-transduced neurosphere cells acquire a glutamatergic identity and give rise to excitatory networks.
(6.10 MB TIF)

Figure S4 **Neuronal reprogramming of postnatal astroglia expanded as neurospheres by forced expression of Neurog2 or Dlx2.** (A–B) Double immunostaining for DsRed (red) and MAP2 (green) reveals that virtually all astroglia-derived neurosphere cells transduced with Dlx2-DsRed (A) or Neurog2-DsRed (B) differentiate into MAP2-positive neurons (14 DPI). (C–D) Fluorescence micrographs at the same magnification showing that astroglia-derived neurosphere cells transduced with Neurog2 (D) extend several MAP2-positive neurites, whereas Dlx2-reprogrammed neurons show more simple dendritic arbors and often exhibit a bipolar morphology (C). Scale bars: A, B: 60 µm; C, D: 20 µm.
(7.52 MB TIF)

Figure S5 **Calcium imaging experiments of postnatal astroglia-derived neurosphere cells transduced with Dlx2 do not reveal any spontaneous Ca^2+^ transients.** (A) Cultures of astroglia-derived neurosphere cells transduced with Dlx2 (red) were incubated at 27 DPI with the calcium-sensitive probe Oregon Green BAPTA1 (green). (B) Micrograph of the same field of view as shown in (A) depicting five astroglia-derived neurosphere cells transduced with Dlx2 exhibiting a neuronal morphology (arrows) that are analysed in (C). (C) The graph shows a virtual absence of any spontaneous Ca^2+^ transient in the five DsRed-positive analysed neurons shown in (B). The trace of each individual neuron is shown in a different colour.
(4.55 MB TIF)

Figure S6 **Adult injured cortex-derived neurosphere cells generate functional GABAergic neurons following forced expression of Dlx2.** (A–A') Micrographs showing an adult cortex-derived neurosphere cell 28 d after transduction with Dlx2 (red) that has differentiated into a MAP2-positive neuron (green). (B–B') Step-depolarisation of the adult cortex-derived neurosphere cell transduced with Dlx2 shown in (B) evokes a GABAergic autapse at 27 DPI (black trace), which is blocked by the GABA_A_ receptor antagonist bicuculline (10 µM, red trace). Scale bars: A, B: 25 µm.
(1.32 MB TIF)

Video S1 **Direct visualisation of efficient reprogramming of astroglia into neurons by Neurog2.** Time-lapse video-microscopy of an astroglia culture derived from GLAST::CreERT2/Z/EG mice following recombination in vivo between P2–P7 and transduced with a retrovirus encoding Neurog2-IRES-DsRed. The right screen reveals those cells which successfully had undergone recombination by GFP fluorescence. GFP fluorescence images were taken every 12 h after imaging had commenced (onset 18 h post-infection). The left screen shows DsRed fluorescence to reveal transduced cells. Images were taken every 6 h. Tracking of each cell was performed with bright field images taken every 4 min (not shown). DsRed- and GFP-double positive cells are marked by the red and green arrows, respectively. The beige crosses marked dead cells. High magnification views of one DsRed/GFP double-positive cell are shown in Video S2. Note the massive, partially still ongoing, reprogramming by 3 d 6 h. Time is indicated in each image.
(4.03 MB MPG)

Video S2 **Direct visualisation of reprogramming of fate-mapped astroglia into neurons.** Temporal sequence of reprogramming by Neurog2 of a fate-mapped astrocyte derived from GLAST::CreERT2/Z/EG mice following recombination in vivo between P2 and P7. The video provides high magnification views of the sequence shown in Video S1. The video starts with a GFP-fluorescence and bright field image at the beginning of the time-lapse experiment. The GFP-positive cell is marked with a red arrow throughout the sequence. The cell is subsequently followed using bright field images. At the point when DsRed fluorescence is detectable, a GFP fluorescence image is shown to demonstrate the co-expression of DsRed and GFP in the same cell. Subsequently DsRed fluorescence images (grey scale) monitor the division of the transduced cell and the gradual metamorphosis of the two daughter cells into neurons. At the end of the imaging sequence a GFP fluorescence image is shown to confirm that the two neurons are reporter-positive. Time is indicated in each image.
(6.14 MB MPG)

Video S3 **Direct visualisation of the astroglia-to-neuron metamorphosis in the absence of cell division over a time period of 5 d.** Temporal sequence of high magnification views of an astrocyte culture following transfection with the retroviral plasmid encoding Neurog2-IRES-DsRed. Prior to the onset of DsRed expression, bright field images are shown to follow a cell subsequently found to be transfected (red arrow). DsRed fluorescence images are used to follow the cell through the reprogramming process. Note that the astroglial cell followed by the red arrow is reprogrammed into a neuron without entering cell division.
(5.22 MB MPG)

## Abbreviations

AP: action potential
DPI: days post infection
GFP: green fluorescent protein
Mash1: mammalian achaete-schute homolog 1
Neurog2: neurogenin-2
vGaT: vesicular GABA transporter
vGluT1: vesicular glutamate transporter 1
