# Supplementary figures and images for: Directing Astroglia from the Cerebral Cortex into Subtype Specific Functional Neurons
Source: PLoS Biol. 2010 May 18;8(5):e1000373. doi: 10.1371/journal.pbio.1000373 (PMC2872647; doi:10.1371/journal.pbio.1000373)

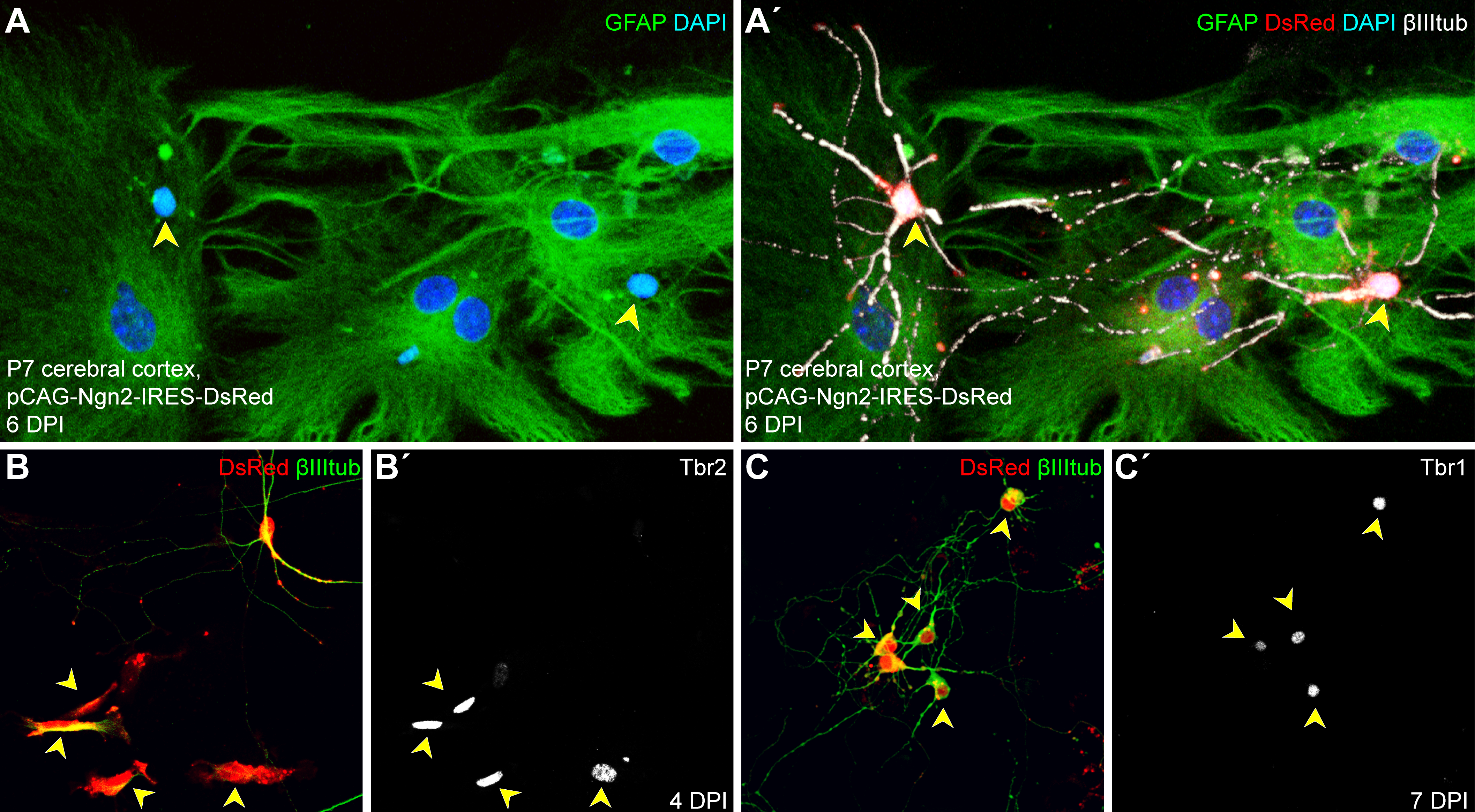

Supplement: Figure S1 — Neuronal reprogramming of astroglia involves down-regulation of GFAP and up-regulation of Tbr2 and Tbr1. (A–A') Down-regulation of GFAP in astroglia-derived neurons. Astroglia culture 6 d post-transduction with Neurog2-IRES-DsRed encoding retrovirus. (A) Note that with the exception of the two cells labelled by the arrowheads depicting DAPI positive nuclei (blue) devoid of any GFAP immunoreactivity, all other cells labelled by nuclear DAPI staining are GFAP-positive (green). (A') The two GFAP-negative cells are DsRed-positive (indicating Neurog2 expression) and are βIII tubulin-positive (white) revealing their neuronal identity (arrowheads). (B–B') Expression of Tbr2 in astroglia undergoing reprogramming at 4 DPI. (B) Immunostaining for DsRed (red) and βIII tubulin (green) shows five cells transduced with Neurog2-IRES-DsRed encoding retrovirus, one of which already developed into a neuron, while in the other cells βIII tubulin expression has barely commenced. (B') Immunostaining for Tbr2 (white, arrowheads) reveals expression in those transduced astroglia which still exhibit glia-like morphology (four out of five cells). This is consistent with the early expression of Tbr2 in the cascade of transcription factors steering glutamatergic neurogenesis [21]. (C–C') Expression of Tbr1 in neurons derived from Neurog2-transduced astroglia at 7 DPI. (C) Immunostaining for DsRed (red) and βIII tubulin (green) reveals five reprogrammed cells. (C') Immunostaining for Tbr1 (white, arrowheads) reveals the up-regulation of Tbr1 by Neurog2 in a large subset (four out of five cells) of Neurog2-transduced cells as reported previously [7]. (7.08 MB TIF) [file pbio.1000373.s001.tif]

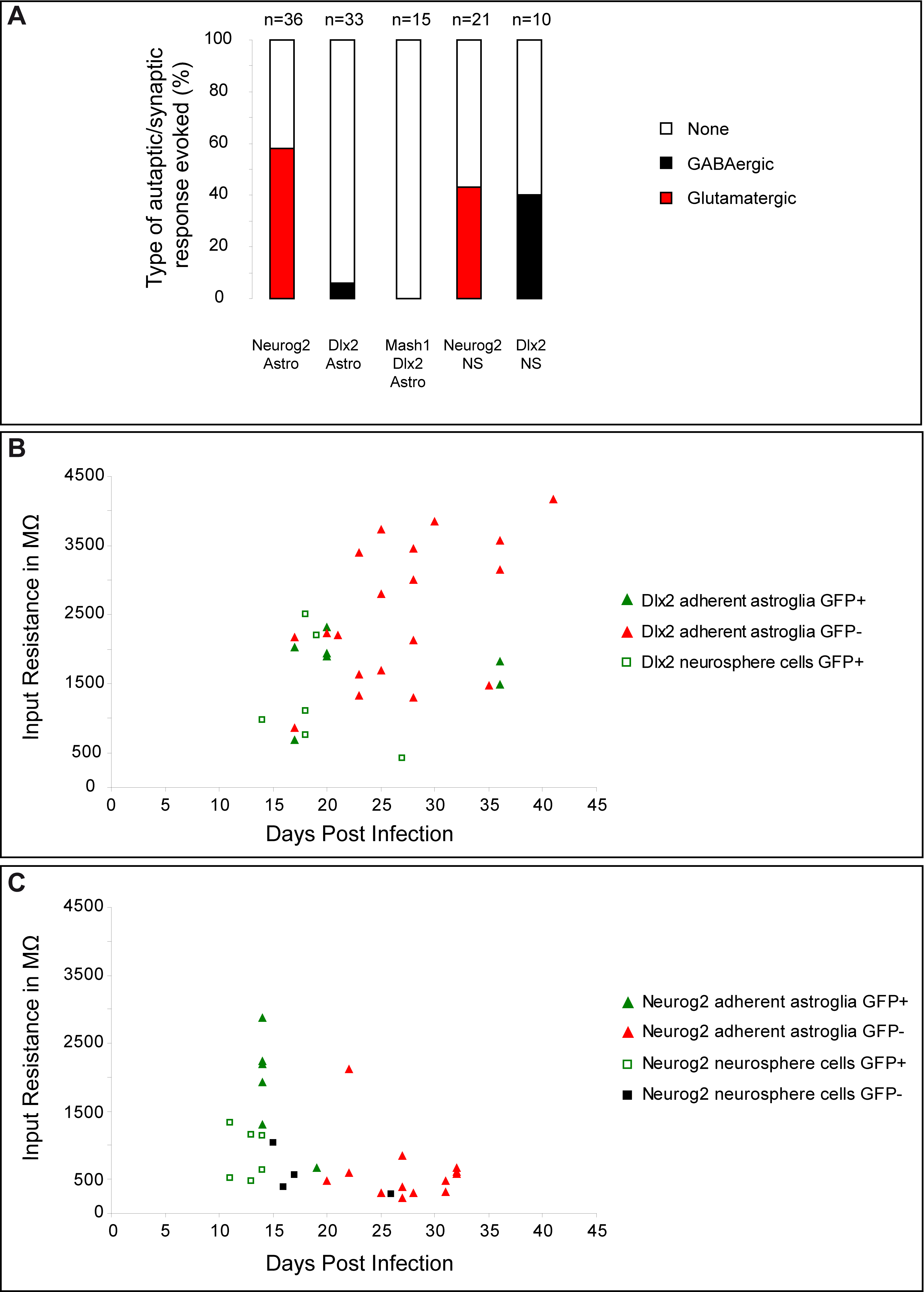

Supplement: Figure S2 — Type of synaptic responses and input resistances of postnatal astroglia-derived neurons reprogrammed by Neurog2 or Dlx2. (A) Summary of the type of autaptic/synaptic responses that could be evoked from astroglia reprogrammed by forced expression of Neurog2, Dlx2, or Mash1 in combination with Dlx2, following culturing under adherent (Astro) or neurosphere conditions (NS). The number of neurons exhibiting autaptic/synaptic connections is expressed as percentage of recorded neurons. Note that astroglia-derived neurons reprogrammed by Neurog2 or Dlx2 in adherent astroglia cultures were recorded at 24.6±0.9 DPI or 26.9±1.4 DPI, respectively. Astroglia-derived neurosphere cells transduced with Neurog2 or Dlx2 were recorded at 14.2±0.7 DPI or 20.1±1.6 DPI, respectively. (B) The graph plots input resistance values over time (MΩ) of astroglia-derived neurons reprogrammed by Dlx2, following culturing under adherent (triangles) or neurosphere conditions (squares), respectively. GFP reporter-positive fate-mapped cells are depicted with green colour. (C) The graph plots input resistance values over time (MΩ) of astroglia-derived neurons reprogrammed by Neurog2, following culturing under adherent (triangles) or neurosphere conditions (squares), respectively. GFP reporter-positive fate-mapped cells are depicted with green colour. (0.45 MB TIF) [file pbio.1000373.s002.tif]

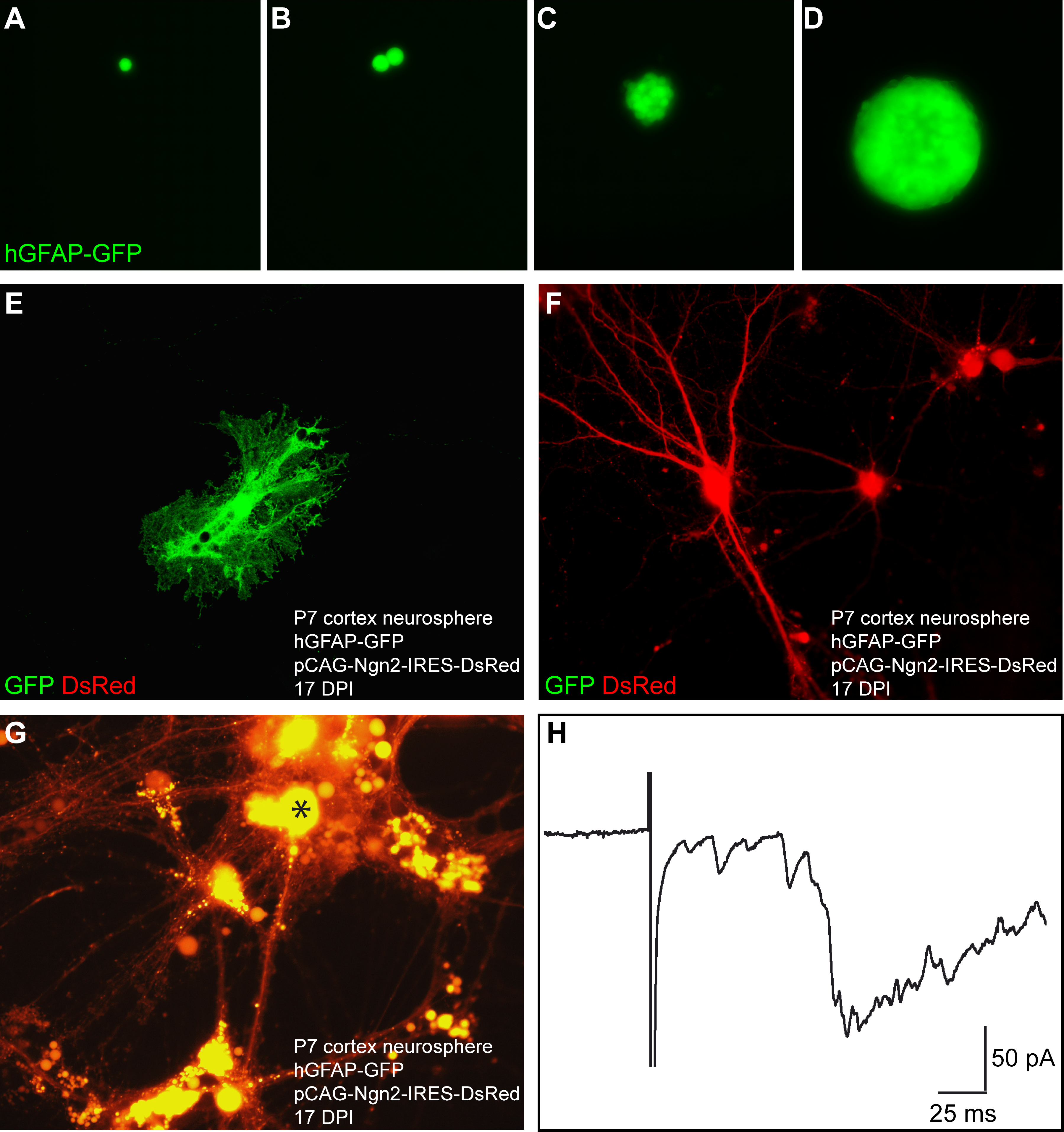

Supplement: Figure S3 — Postnatal astroglia isolated from hGFAP-GFP mice and expanded as neurospheres generate glutamatergic neurons following forced expression of Neurog2. (A) To ascertain the astroglial origin of the neurosphere founder cells, cortical tissue from P5–P7 hGFAP-GFP mice was cultured as single cell in Terasaki plates. (B) Fluorescence micrograph depicting cell division of a singly plated GFP-positive astroglial cell. (C–D) The majority of singly plated GFP-positive astroglia gives rise to GFP-positive neurospheres (C) that increase in size over time in culture (D). (E–F) Double immunostaining for DsRed (red) and GFP (green) of astroglia-derived neurospheres as shown in (D), 17 d after neurosphere dissociation and subsequent transduction with Neurog2-IRES-DsRed. (E) Example of a non-transduced cell (i.e., DsRed-negative) expressing GFP and exhibiting a glial morphology. (F) In contrast, following transduction with Neurog2-DsRed, neurosphere cells lose GFP expression and differentiate into neurons. (G) The fluorescence micrograph depicts a cluster of Neurog2-expressing neurosphere cells, derived from singly plated GFP-positive astroglia that lost GFP expression and exhibit a neuronal morphology 17 DPI. (H) The graph shows an evoked sequence of both autaptic and polysynaptic events following step-depolarisation of the neuron marked by the asterisk in (G), suggesting that Neurog2-transduced neurosphere cells acquire a glutamatergic identity and give rise to excitatory networks. (6.10 MB TIF) [file pbio.1000373.s003.tif]

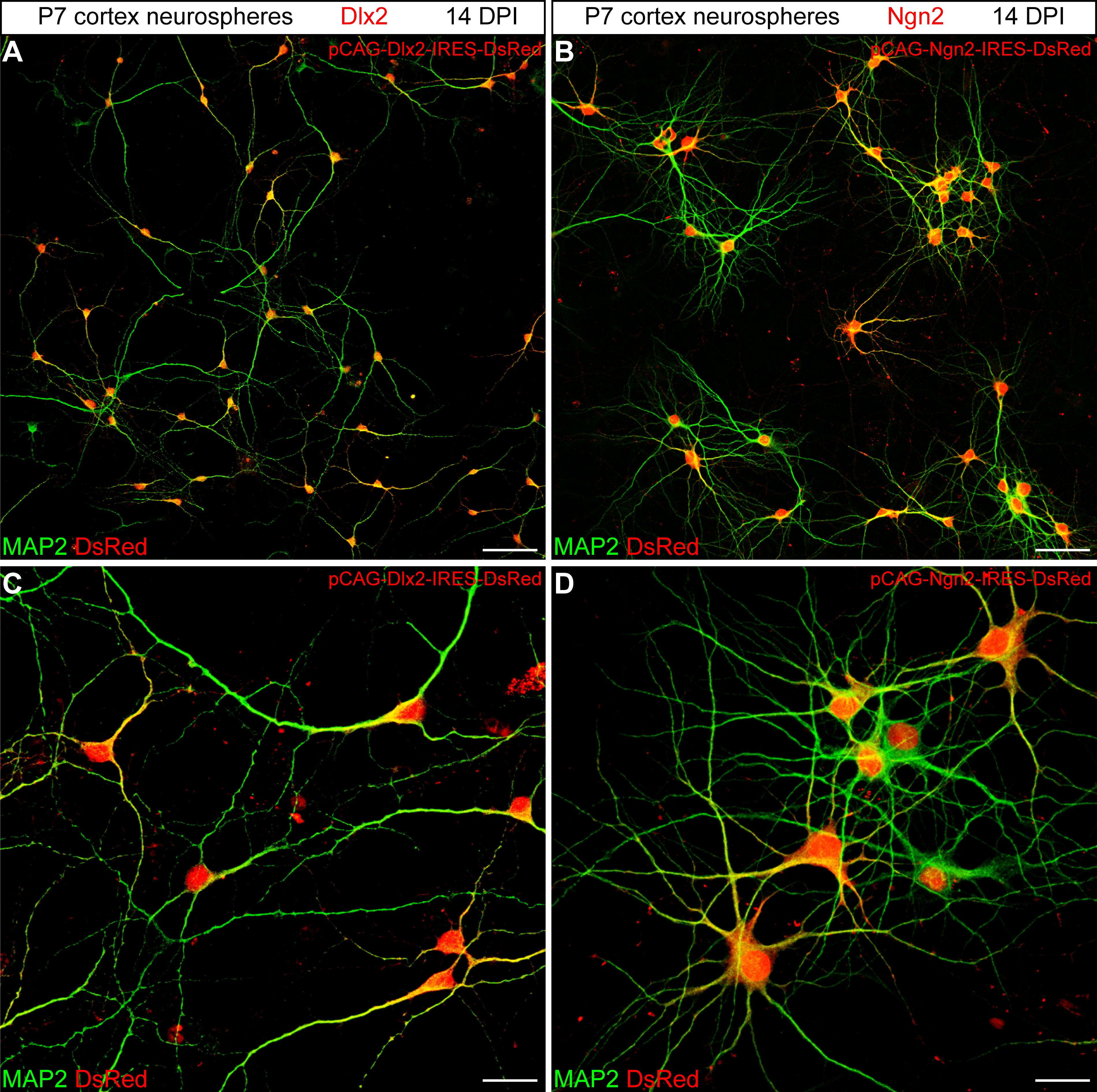

Supplement: Figure S4 — Neuronal reprogramming of postnatal astroglia expanded as neurospheres by forced expression of Neurog2 or Dlx2. (A–B) Double immunostaining for DsRed (red) and MAP2 (green) reveals that virtually all astroglia-derived neurosphere cells transduced with Dlx2-DsRed (A) or Neurog2-DsRed (B) differentiate into MAP2-positive neurons (14 DPI). (C–D) Fluorescence micrographs at the same magnification showing that astroglia-derived neurosphere cells transduced with Neurog2 (D) extend several MAP2-positive neurites, whereas Dlx2-reprogrammed neurons show more simple dendritic arbors and often exhibit a bipolar morphology (C). Scale bars: A, B: 60 µm; C, D: 20 µm. (7.52 MB TIF) [file pbio.1000373.s004.tif]

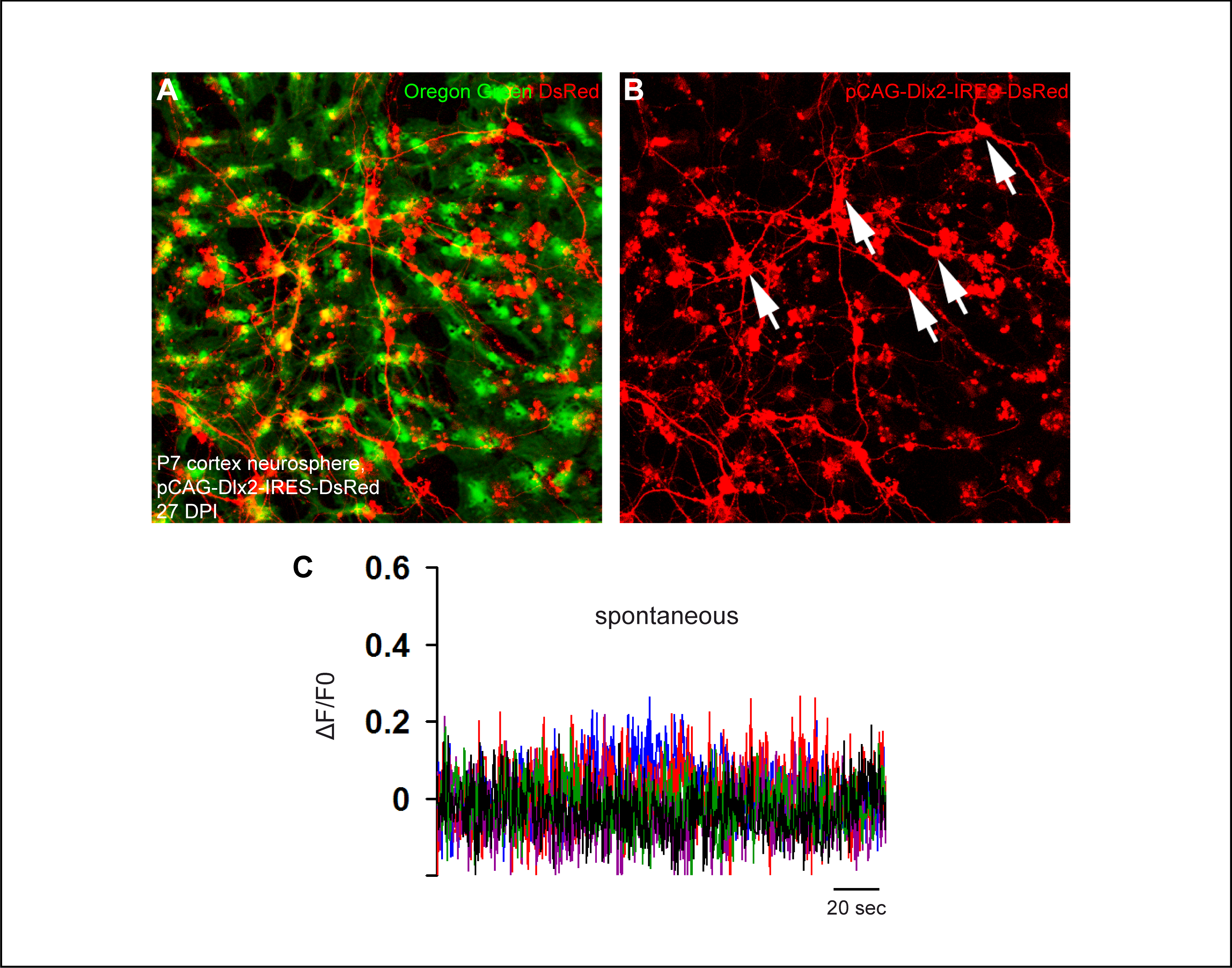

Supplement: Figure S5 — Calcium imaging experiments of postnatal astroglia-derived neurosphere cells transduced with Dlx2 do not reveal any spontaneous Ca2+ transients. (A) Cultures of astroglia-derived neurosphere cells transduced with Dlx2 (red) were incubated at 27 DPI with the calcium-sensitive probe Oregon Green BAPTA1 (green). (B) Micrograph of the same field of view as shown in (A) depicting five astroglia-derived neurosphere cells transduced with Dlx2 exhibiting a neuronal morphology (arrows) that are analysed in (C). (C) The graph shows a virtual absence of any spontaneous Ca2+ transient in the five DsRed-positive analysed neurons shown in (B). The trace of each individual neuron is shown in a different colour. (4.55 MB TIF) [file pbio.1000373.s005.tif]

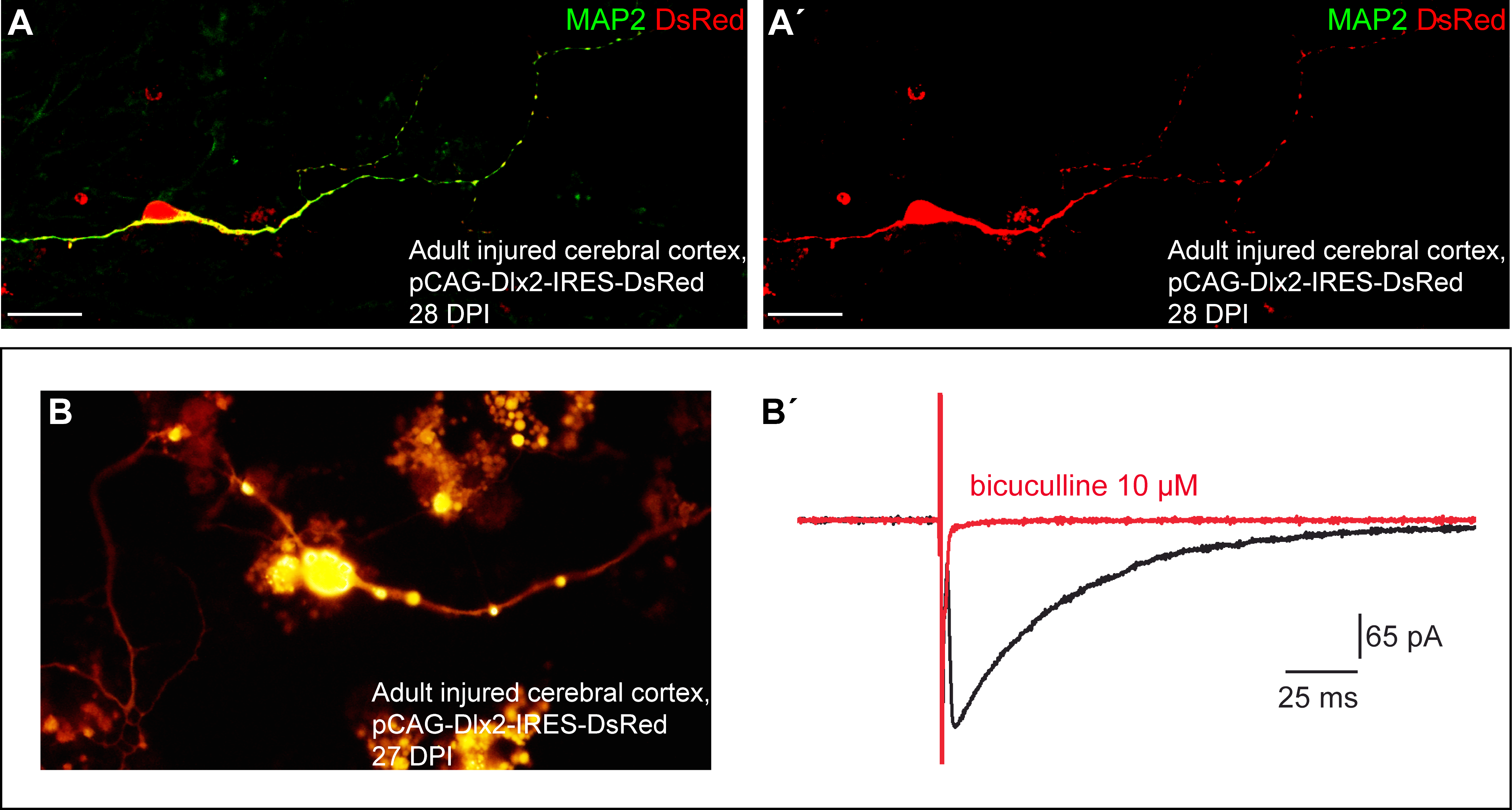

Supplement: Figure S6 — Adult injured cortex-derived neurosphere cells generate functional GABAergic neurons following forced expression of Dlx2. (A–A') Micrographs showing an adult cortex-derived neurosphere cell 28 d after transduction with Dlx2 (red) that has differentiated into a MAP2-positive neuron (green). (B–B') Step-depolarisation of the adult cortex-derived neurosphere cell transduced with Dlx2 shown in (B) evokes a GABAergic autapse at 27 DPI (black trace), which is blocked by the GABAA receptor antagonist bicuculline (10 µM, red trace). Scale bars: A, B: 25 µm. (1.32 MB TIF) [file pbio.1000373.s006.tif]
